# Study quality and efficacy of psychological interventions for posttraumatic stress disorder: a meta-analysis of randomized controlled trials

**DOI:** 10.1017/S0033291721001641

**Published:** 2021-06

**Authors:** Nexhmedin Morina, Thole H. Hoppen, Ahlke Kip

**Affiliations:** Institute of Psychology, University of Münster, Münster, Germany

**Keywords:** Meta-analysis, PTSD, study quality, treatment

## Abstract

**Background:**

Research indicates that higher study quality may be associated with smaller treatment effects. Yet, knowledge about the association between study quality and treatment efficacy for posttraumatic stress disorder (PTSD) is limited. We aimed at evaluating the efficacy of psychological interventions for adult PTSD and the association between study quality and treatment effects.

**Methods:**

We conducted a systematic search to identify randomized controlled trials (RCTs) that examined the efficacy of psychological interventions for chronic PTSD symptoms in adult samples with at least 70% of patients being diagnosed with PTSD by means of a structured interview. We assessed study quality using the following eight criteria from prior research: *N* ⩾ 50, all patients met criteria for PTSD, a treatment manual was used, therapists were trained, treatment integrity was checked, intent-to-treat analyses were applied, randomization was conducted by an independent party, and treatment outcome was conducted by blind assessors.

**Results:**

The search resulted in 136 RCTs with 8978 patients. Active treatment conditions were largely effective in reducing PTSD symptoms at posttreatment and follow-up (Hedges' *g* = 1.09 and 0.81, respectively) when compared to passive control conditions. The comparison to active control conditions at posttreatment and follow-up resulted in medium effect sizes. A total of 14 trials met all study quality criteria and these trials produced large effect sizes when compared to passive control conditions at posttreatment and follow-up.

**Conclusions:**

Overall, study quality was not significantly associated with effect size. The findings indicate that psychological interventions can effectively reduce PTSD symptoms irrespective of study quality.

## Introduction

Posttraumatic stress disorder (PTSD) is a prevalent condition with a chronic course if untreated (Kessler et al., 2017; Morina, Wicherts, Lobbrecht, & Priebe, 2014). Several psychological interventions have been developed to treat this disorder and a large amount of clinical trials has investigated their efficacy. Meta-analytic reviews have concluded that psychological interventions for adult PTSD produce large effect sizes (e.g. Bisson, Roberts, Andrew, Cooper, & Lewis, 2013; Cusack et al., 2016). However, there is lack of knowledge about the association of study quality and treatment efficacy. Literature on the efficacy of treatments for depression indicates that trials with low study quality have overestimated treatment efficacy for both psychopharmacology (Kirsch et al., 2008; Turner, Matthews, Linardatos, Tell, & Rosenthal, 2008) as well as psychological treatment (Cuijpers, van Straten, Bohlmeijer, Hollon, & Andersson, 2010; Gellatly et al., 2007; Klein, Jacobs, & Reinecke, 2007; Weisz, McCarty, & Valeri, 2006). Following a thorough examination of the relationship between study quality and treatment efficacy, Cuijpers et al. (2010) concluded that the efficacy of psychological interventions for adult depression has been overestimated in the past. In this study, the authors assessed eight characteristics of study quality that were based on the criteria for assessing the quality of treatment delivery originally recommended by Chambless and Hollon (1998) as well as on the criteria proposed by the Cochrane Collaboration to assess the methodological validity of a study (Higgins & Green, 2009). These criteria dictate that primary complaints were assessed with a valid diagnostic interview, a treatment manual was used, therapists were trained, treatment integrity was assessed, intent-to-treat (ITT) analyses were conducted, at least 50 participants were treated in the active and control group, an independent party conducted randomization, and assessors were blinded. Cuijpers et al. (2010) concluded that only 11 out of 115 randomized clinical trials for depression were of high quality and that these trials had a significantly lower mean effect size than the other trials (*d* = 0.22 and 0.74, respectively). The small effect size of 0.22 found in high-quality trials for depression is alarming and calls for a thorough investigation of the role of trial quality in treatment efficacy. Prior to that, two meta-analyses on the efficacy of psychological interventions for pediatric depression had also found evidence that the effects of psychotherapy for depression have been overestimated (Klein et al., 2007; Weisz et al., 2006).

Meta-analytic reviews of treatment efficacy for PTSD have applied different criteria to assess potential risks of bias and have generally concluded that methodological quality varied considerably across the trials and that risk of bias was high or unclear in a large proportion of older studies (Bisson et al., 2013; Cusack et al., 2016). Gerger, Munder, and Barth (2014) meta-analyzed 18 trials comparing the efficacy of specific *v.* unspecific psychological interventions for adult PTSD and concluded that high-quality trials (*k* = 4) did not significantly differ from lower-quality trials (*k* = 14). However, these results are limited by the low number of included trials and the low number of applied criteria to assess study quality (i.e. concealment of treatment allocation, adequacy of statistical analyses, and adequacy of outcome assessment).

We still lack a thorough analysis of the relationship between study quality and treatment efficacy for adult PTSD. Therefore, we aimed at providing a quantitative, meta-analytic review of the efficacy of interventions for adults suffering from PTSD and at investigating the relationship between study quality and treatment efficacy. For this purpose, we reviewed randomized controlled trials (RCTs) that compared the efficacy of psychological interventions relative to active control conditions (ACC) or passive control conditions (PCC). We first tested the hypothesis that psychological interventions can effectively reduce symptoms of PTSD. To this end, we examined the efficacy of active treatment conditions relative to PCC and ACC at posttreatment and at follow-up. Furthermore, we expected study quality to be negatively associated with treatment efficacy. We assessed the potential association between study quality and effect sizes by (1) comparing effect-sizes of high-quality *v.* lower-quality trials, (2) by examining study quality as a continuous predictor of effect sizes, and (3) by examining the relationship between the eight individual quality criteria and effect sizes. We defined the main structured research question describing the Population, Intervention, Comparison, Outcome, and Study design in accordance with the recommendations by the Preferred Reporting Items for Systematic Reviews and Meta-analysis (PRISMA) group (Moher, Liberati, Tetzlaff, & Altman, 2009). The question was ‘In patients with PTSD (P), does psychological treatment (I), compared to control conditions (C), improve PTSD (O) in randomized controlled trials (S)?’

## Method

The aims and methods of this meta-analysis were registered with the PROSPERO database (CRD42018094698, http://www.crd.york.ac.uk/prospero).

### Identification and selection of studies

The current systematic review was first based on the literature included in the meta-analysis by Bisson et al. (2013). Then, we conducted a systematic search in the databases PsycINFO and Medline for the period between 1 January 2013 and 22 September 2020. Finally, we reviewed the relevant meta-analyses on the efficacy of psychological interventions for adult PTSD published since 2013 (Asmundson et al., 2019; Barawi, Lewis, Simon, & Bisson, 2020; Bisson, van Gelderen, Roberts, & Lewis, 2020; Carpenter et al., 2018; Cipriani et al., 2018; Coventry et al., 2020; Cusack et al., 2016; Gallegos, Crean, Pigeon, & Heffner, 2017; Gerger et al., 2014; Grasser & Javanbakht, 2019; Hegberg, Hayes, & Hayes, 2019; Hopwood & Schutte, 2017; Karatzias et al., 2019; Khan et al., 2018; Kline, Cooper, Rytwinksi, & Feeny, 2018; Kuester, Niemeyer, & Knaevelsrud, 2016; Lenz, Haktanir, & Callender, 2017; Lewis, Roberts, Andrew, Starling, & Bisson, 2020a; Lewis, Roberts, Bethell, Robertson, & Bisson, 2018; Lewis, Roberts, Gibson, & Bisson, 2020b; Mahoney, Karatzias, & Hutton, 2019; Mavranezouli et al., 2020; Montero-Marin, Garcia-Campayo, López-Montoyo, Zabaleta-Del-Olmo, & Cuijpers, 2018; Morina, Malek, Nickerson, & Bryant, 2017a; Niles et al., 2018; Schwartze, Barkowski, Strauss, Knaevelsrud, & Rosendahl, 2019; Springer, Levy, & Tolin, 2018; Tran & Gregor, 2016; Van Dis et al., 2020; Wilson et al., 2018). Inclusion criteria for the meta-analysis were: (1) RCT, (2) treatment targets primarily chronic PTSD, (3) participants older than 17 years, (4) at least ten participants per condition at post-assessment, and (5) at least 70% of the sample was diagnosed with PTSD by means of a structured interview (Bisson et al., 2013). There were no restrictions on language. Trials on comorbid PTSD and substance use disorders or traumatic brain injury were excluded. Other forms of comorbidity were allowed, yet PTSD needed to be the primary diagnosis.

We conducted multi-field searches (in titles, abstracts, and key concepts) using the following terms (Morina, Koerssen, & Pollet, 2016): *Posttraumatic Stress Disorder* (*Posttraumatic stress* OR *post-traumatic stress* OR Posttraumatic syndrome* OR *PTSD* OR *PTSS*), and *Treatment* (treatment* OR intervention* OR *therapy* OR *psychotherapy* OR *exposure* OR *trial* OR *counselling*). Two independent investigators first inspected the title and abstract of all hits and then read full texts of the hits that seemed to meet the aforementioned inclusion criteria.

### Quality assessment

To code for the quality of included studies, we applied the eight quality criteria used by Cuijpers et al. (2010): (1) participants met diagnostic criteria for PTSD identified with a diagnostic interview, (2) use of a treatment manual, (3) training of therapists, (4) assessment of treatment integrity, (5) report of ITT analyses, (6) at least 50 patients were included in a comparison, (7) independent randomization, and (8) blinded assessors. The criteria were coded categorically with ‘1’ if the criterion was fulfilled or ‘0’ if the criterion was not met or not reported (Cuijpers et al., 2010). Accordingly, a trial receiving eight points was rated as being of high quality. The second and third authors coded the quality criteria independently and discrepancies were resolved in joint discussions with the first author.

### Coding of trial characteristics

Two independent investigators coded and extracted from each study: comparison group(s), number of participants, type of outcome measure, intervention format (individual or group), number and length of sessions, length of follow-up, age of participants, percentage of participants with a diagnosis of PTSD at pretreatment, female gender, type of intervention, country where the trial was conducted, type of traumatic event, and outcome scores (mean and standard deviation). If a publication reported more than one outcome measure of PTSD, we prioritized clinician-based PTSD measures (e.g. Blake et al., 1995) over self-reports. Furthermore, publications that exclusively reported a comparison between two active interventions belonging to the same treatment family (e.g. Acierno et al., 2017) were not included. The follow-up period was divided into follow-up 1 (FU1) assessed up to 20 weeks after posttreatment and follow-up 2 (FU2) assessed more than 20 weeks after posttreatment. In line with previous meta-analyses on interventions for PTSD (Morina et al., 2016; Morina, Malek, Nickerson, & Bryant, 2017b), treatment interventions were first coded as either active treatment or control group. The active treatment group was then subdivided into trauma-focused cognitive-behavior therapy (TF-CBT), eye movement desensitization and reprocessing (EMDR), or other active treatment conditions (i.e. interpersonal psychotherapy, imagery rescripting, present centered therapy, meta-cognitive therapy, emotion focused supportive psychotherapy, dialogical exposure therapy, dialectical behaviour therapy, and mindfulness-based interventions). The control groups were subdivided into PCC, consisting of waitlist, single session psychoeducation, minimal attention, and self-administered relaxation interventions, and ACC, consisting of supportive counseling, treatment as usual, medical placebo, active listening, guided psychoeducation, stress inoculation training, self-help booklets, and guided relaxation training.

### Statistical analysis

Data from ITT samples were used when available (72 publications) and completer samples were utilized if ITT samples were not provided (52 publications). To calculate an effect size, the control group mean was subtracted from the treatment group mean at posttreatment or follow-up, respectively, and divided by the pooled standard deviation. Subsequently, the outcome was multiplied by a sample size correction factor *J* = 1 − (3/(4df − 1)) to obtain the effect size Hedges' *g* (Lipsey & Wilson, 2009). Subgroup analyses were conducted if a specific group of interventions consisted of at least four comparisons (Morina et al., 2016). Analyses were completed with the metafor package (v.1.9.8) in R 3.5. using random-effect models given the heterogeneity of the studies (R Core Team, 2015; Viechtbauer, 2010). Effect sizes may be conservatively interpreted with Cohen's convention of small (0.2), medium (0.5), and large (0.8) effects (Cohen, 2013). To examine heterogeneity of effect sizes, we calculated the *Q*-statistic and the *I*^2^-statistic that is an indicator of heterogeneity in percentages, with higher percentages indicating high heterogeneity. The variance of true effect (*τ*^2^) was assessed. To consider potential inequality in effect sizes, prediction intervals (PIs) were calculated using *τ*^2^. PIs estimate an interval within which the estimate is expected to be (IntHout, Ioannidis, Rovers, & Goeman, 2016; Riley, Higgins, & Deeks, 2011). We also calculated the numbers needed to treat (NNT), which inform about the numbers of patients needed to be treated to prevent one adverse event and is more easily interpretable from a clinical perspective (Kraemer & Kupfer, 2006). Unlike Cuijpers et al. (2010) who applied a *p* value of 0.10 as the threshold for statistical significance, we utilized the more conservative *p* value of 0.05.

Potential publication bias was assessed through visual inspection of funnel plots for the primary outcome measures and analyses including more than nine comparisons (Sterne et al., 2011). Furthermore, we calculated the likely number of missing studies using the trim and fill procedure, which yields an estimate of the effect size after publication bias has been taken into account (Duval & Tweedie, 2000). The association between quality criterion and treatment efficacy was examined by factorial subgroup analyses, whereas the overall influence of quality was investigated with mixed-effect models. If outliers emerged, we repeated the respective analysis without the outliers. We defined an effect size as an outlier when it was at least 3.3 standard deviations below or above the pooled mean effect size (Hunter & Schmidt, 2007; Tabachnick & Fidell, 2013).

## Results

### Selection and characteristics of included studies

**Fig. 1.**
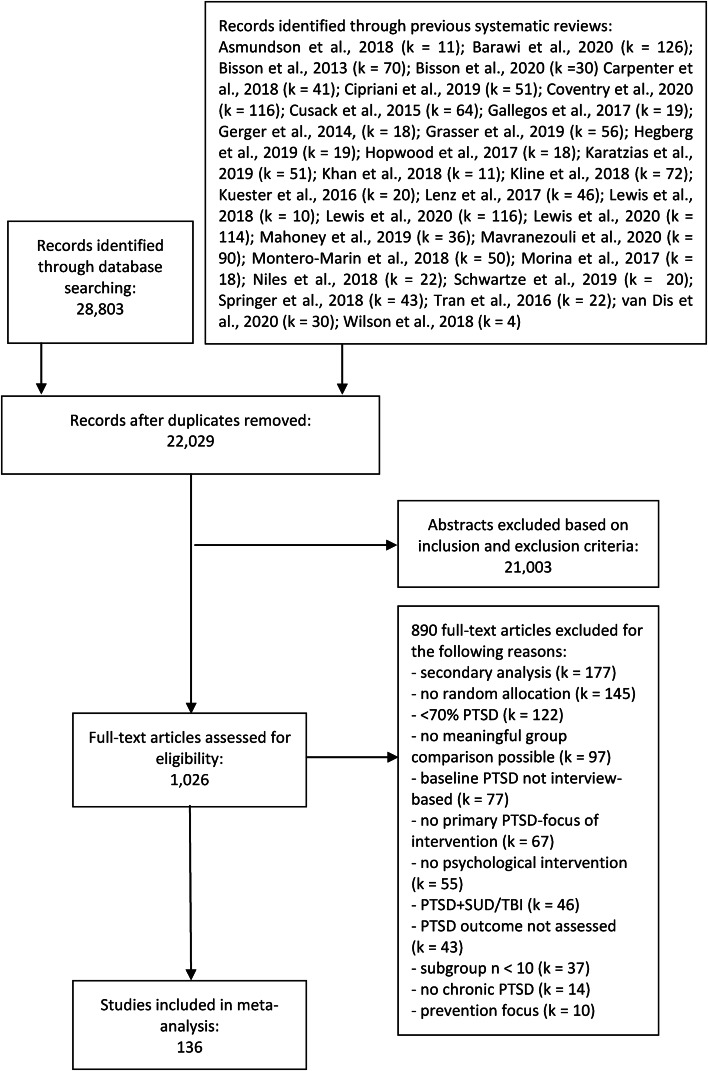
Flowchart of study selection.

Figure 1 displays a PRISMA (Moher et al., 2009) flow diagram of the publication selection process. After examining 22 029 abstracts, 1026 full text publications were reviewed. The final review resulted in 136 clinical trials with 8978 participants in total (5695 in an active treatment condition and 3283 in a PCC or ACC).

Relevant characteristics of the 136 trials are summarized in the online Supplementary material (Appendix A & B). All publications were journal articles in English, 119 of the trials were conducted in a Western country (four with refugees), while 17 were conducted in non-Western countries (four with refugees). The mean age of participants was 40.04 years (s.d. = 8.60) and 54.67% of them were female. Participants entered treatment on the basis of PTSD symptoms resulting from different traumatic events, with 65 publication including participants with diverse trauma backgrounds and combat being the most common form of specific traumatic experience. Individual treatment was applied in 73.53% of the trials (see Appendix A).

Appendix D in the online Supplementary material describes the number of active treatment and control conditions being examined in the included studies. Relevant follow-up data (i.e. at least one relevant group comparison available) were reported for 64 active treatment conditions. Some publications did not provide controlled follow-up data (e.g. Asukai, Saito, Tsuruta, Kishimoto, and Nishikawa, 2010) and two trials (Neuner *et al*. 2010; Orang *et al*. 2018) did not report controlled posttreatment data. In one trial, the subgroup sample size was above 9 only for the follow-up measures and this trial was included only in follow-up comparison (Krupnick et al., 2008).

### Treatment efficacy

In total, 65 and 53 publications reported on the efficacy of 77 and 59 active treatment conditions compared to PCC and ACC at posttreatment, respectively. The pooled effect sizes of these comparisons were significantly larger (*p* < 0.001) when using PCC (*g* = 1.08) than using ACC (*g* = 0.47; see Table 1). Yet, overall the results demonstrate that psychological interventions are significantly more effective in reducing PTSD symptoms than both PCC and ACC. Heterogeneity was large for comparisons to both PCC and ACC at posttreatment, respectively (*I*^2^ = 81.22; *Q* = 318.12, *p* < 0.001 and *I*^2^ = 57.23; *Q* = 133.37, *p* < 0.001), indicating substantial heterogeneity in effect sizes between studies. Four trials comparing active treatment conditions to PCC were considered as outliers (Paunović, 2011; Sloan, Marx, Bovin, Feinstein, & Gallagher, 2012; Wells, Walton, Lovell, & Proctor, 2015; Zang, Hunt, & Cox, 2014) and when these were excluded, active conditions (i.e. *k* = 73) still reached an effect size of *g* = 0.99 [95% confidence interval (CI) = 0.86–1.13]. The comparison of active treatment conditions to ACC did not reveal any outliers. As can be seen in Table 1, active treatment conditions produced medium-to-large effect sizes when compared to PCC both up to 20 weeks (i.e. FU1) as well as more than 20 weeks following treatment (i.e. FU2).

**Table 1.** Efficacy of psychological interventions

Post							FU						
	*k*	*g*	s.e.	95% CI (PI)	*I*^2^	NNT	FU1 *v*. FU2	*k*	*g*	s.e.	95% CI (PI)	*I*^2^	NNT
Overall outcomes													
All active conditions *v*. PCC	77	1.09***	0.08	0.93–1.25 (−0.13 to 2.31)	81.22***	1.79	1	21	0.81***	0.12	0.56–1.05 (−0.15 to 1.77)	73.47***	2.32
							2	8	0.67***	0.08	0.51–0.83 (0.47–0.88)	8.14	2.73
All active conditions *v*. ACC	59	0.47***	0.06	0.35–0.58 (−0.17 to 1.11)	57.23***	3.86	1	30	0.47***	0.09	0.31–0.64 (−0.22 to 1.17)	57.26***	3.80
							2	19	0.78***	0.12	0.54–1.03 (−0.09 to 1.65)	68.09***	2.38
Subgroup analyses													
TF-CBT *v*. PCC	47	1.23***	0.12	1.00–1.47 (−0.24 to 2.71)	85.90***	1.62	1	13	0.89***	0.17	0.56–1.22 (−0.18 to 1.97)	77.43***	2.12
							2	6	0.75***	0.09	0.57–0.93 (0.56–0.95)	2.80	2.47
TF-CBT *v*. ACC	33	0.49***	0.08	0.33–0.66 (−0.24 to 1.23)	61.71***	3.68	1	20	0.57***	0.10	0.38–0.76 (−0.02 to 1.16)	45.03*	3.19
							2	17	0.79***	0.14	0.52–1.06 (−0.15 to 1.73)	70.21***	2.36
TF-CBT *v*. OAC (excluding EMDR)	19	0.08	0.06	−0.03 to 0.20 (0.04–0.22)	33.47*	20.94	1	11	0.22*	0.09	0.05–0.39 (−0.21 to 0.64)	53.60*	8.22
							2	11	0.15**	0.06	0.04–0.26 (0.04–0.26)	0.00	11.65
TF-CBT *v*. EMDR	10	−0.06	0.21	−0.48 to 0.35 (−1.21 to 1.09)	70.79**	−28.97	1	4	−0.12	0.30	−0.72 to 0.47 (−1.18 to 0.94)	54.29	−14.32
							2	n.a. (*k* = 2)					
EMDR *v*. PCC	7	1.19***	0.22	0.76–1.62 (0.16–2.22)	71.86***	1.67	1 & 2	n.a. (*k* = 2); n.a. (*k* = 1), respectively					
EMDR *v*. ACC	11	0.42***	0.10	0.22–0.62 (0.15–0.68)	7.31	4.29	1 & 2	n.a. (*k* = 3); n.a. (*k* = 0), respectively					
Other active conditions (OAC) *v*. PCC	21	0.76***	0.08	0.61–0.91 (0.36–1.16)	30.33**	2.45	1	4	0.41**	0.13	0.14–0.67 (0.14–0.67)	0.00	4.39
							2	n.a. (*k* = 1)					
Other active conditions (OAC) *v*. ACC	15	0.53***	0.13	0.27–0.79 (−0.36 to 1.42)	73.77***	3.42	1	7	0.49	0.21	0.09–0.90 (−0.50 to 1.49)	73.56**	3.67
							2	n.a. (*k* = 2)					

ACC, active control conditions; *k,* number of trials included in the analysis for the given comparison; n.a., number of trials too small (*k* < 4) to conduct analysis; EMDR, eye movement desensitization and reprocessing; FU, follow-up; OAC, other active conditions; PCC, passive control conditions; PI, prediction interval; TF-CBT, trauma-focused Cognitive behavior therapy; WL, waitlist. For outlier- and asymmetry-adjustments see Appendix D.

*p < 0.05, **p < 0.01, ***p < 0.001.

Subgroup analyses at posttreatment revealed that TF-CBT interventions produced large and medium effect sizes when compared to PCC and ACC (see Table 1). Similar effect sizes were also found for EMDR when compared to PCC and ACC. Other active treatment conditions (i.e. excluding TF-CBT and EMDR) also produced large and medium effect sizes when compared to PCC and ACC. A comparison of TF-CBT to other active treatment conditions (excluding EMDR) and to EMDR revealed nonsignificant effect sizes.

### Publication bias

With respect to comparisons with PCC, the visual inspection of the funnel plot and Egger's test pointed at significant asymmetry of the funnel plot (*p* < 0.001), but Duval and Tweedie's trim and fill procedure did not indicate missing trials. With regard to comparisons with ACC, the visual inspection of the funnel plot and Egger's test also indicated significant asymmetry of the funnel plot (*p* = 0.001). Duval and Tweedie's trim and fill procedure indicated four missing trials and the adjusted effect size was *g* = 0.42, 95% CI = 0.30–0.54. Note that this represents a small change in effect size (i.e. from 0.47 to 0.42).

### Study quality

**Fig. 2.**
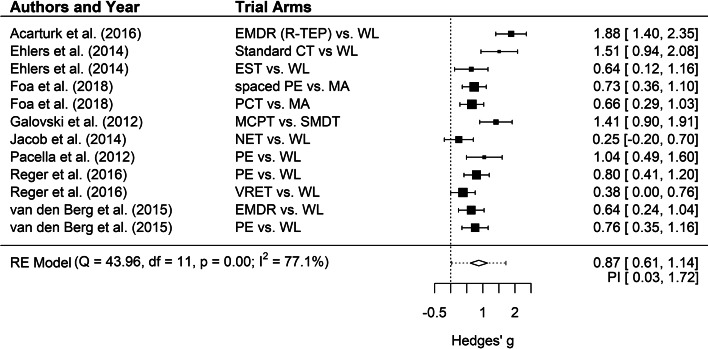
Effect sizes of high-quality trials on treatments for PTSD compared to passive control conditions at post-test.

Intraclass correlation coefficient of the total score for all studies combined among the two raters of study quality was 0.79, 95% CI = 0.76–0.81, indicating good inter-rater reliability. Overall, study quality was moderate with a mean of 5.82 (s.d. = 1.48). The vast majority of trials (91.91%) received at least half of the quality scores. A total of 14 trials (10.29%) met all eight quality criteria (Acarturk et al., 2016; Bohus et al., 2020; Cloitre et al., 2010; Ehlers et al., 2014; Foa et al., 2018; Galovski, Blain, Mott, Elwood, & Houle, 2012; Ivarsson et al., 2014; Jacob, Neuner, Maedl, Schaal, & Elbert, 2014; Langkaas et al., 2017; Pacella et al., 2012; Reger et al., 2016; Sloan, Unger, Lee, & Beck, 2018; Van den Berg et al., 2015; Van Dis et al., 2007). Eight of these trials compared a total of 12 active treatment conditions to passive control groups and this comparison produced an effect size of *g* = 0.87 (see Fig. 2), which was not significantly smaller than the effect size of the 65 trials with lower quality (*g* = 1.14; see Table 2). Four of the high-quality trials compared active treatment conditions to PCC at FU1 and produced an effect size of *g* = 0.78, CI 0.22–1.33; NNT = 2.40. Only two of the high-quality trials provided FU2 data. Heterogeneity was large for both high- and low-quality trials. Three high-quality trials compared active treatment conditions to ACC (Cloitre et al., 2010; Ivarsson et al., 2014; Van der Kolk et al., 2007) and three high-quality trials compared different active treatments (Bohus et al., 2020; Langkaas et al., 2017; Sloan et al., 2018), precluding meta-analytic review due to small number of available trials.

**Table 2.** Comparison of high-quality (HQ) and lower-quality trials (Other) when compared to passive control conditions at posttreatment

			*k*	*g*	95% CI (PI)	*I*^2^	NNT	*p*
High quality (HQ)		HQ	12	0.87***	0.61–1.14 (0.03–1.72)	77.05***	2.16	0.233
		Other	65	1.14***	0.96–1.33 (−0.19 to 2.47)	81.34***	1.72	
CAPS only		HQ	8	0.78***	0.48–1.07 (0.03–1.52)	70.75**	2.40	0.098
		Other	32	1.21***	0.96–1.45 (−0.05 to 2.47)	80.44***	1.65	
Type of treatment	TF-CBT	HQ	8	0.83***	0.54–1.13 (0.08–1.59)	71.47**	2.25	0.119
		Other	39	1.33***	1.05–1.61 (−0.28 to 2.94)	85.77***	1.53	
	EMDR	HQ	n.a. (*k* = 2)					n.a.
		Other	5	1.16***	0.70–1.61 (0.29–2.02)	52.97	1.70	
	OAC	HQ	n.a. (*k* = 2)					n.a.
		Other	19	0.78	0.60–0.97 (0.24–1.32)	42.93**	2.38	
Treatment format	Individual	HQ	12	0.87***	0.61–1.14 (0.03–1.72)	77.05***	2.16	0.128
		Other	51	1.24***	1.02–1.47 (−0.21 to 2.70)	83.27***	1.61	
	Group	HQ	n.a. (*k* = 0)					n.a.
		Other	14	0.84***	0.56–1.11 (−0.03 to 1.70)	65.97***	2.24	
			*k*	*Intercept*	*b*	*I^2^*	*p*	
Number of sessions as a continuous variable		HQ	12	0.79	0.01	79.40***	0.928	
		Other	65	1.36	−0.02	81.41***	0.209	

CAPS, clinician-administered PTSD scale; *k,* number of trials included in the analysis for the given comparison; n.a., not applicable [i.e. number of trials too small (*k* < 4) to conduct analysis]; OAC, other active conditions; PCC, passive control conditions; PI, prediction interval; TF-CBT, trauma-focused cognitive behavior therapy. *p* values refer to the comparison of high quality *v*. other studies and to the significance level of *b*. For outlier adjustments see Appendix E.

*p < 0.05; **p < 0.01 ***p < 0.001.

We further conducted a series of subgroup analyses to examine whether the following factors were associated with study quality: use of the Clinician-Administered PTSD Scale for DSM (Blake et al., 1995) as treatment outcome, type of treatment (TF-CBT *v.* other), treatment format (individual *v.* group), type of control (PCC *v.* ACC), and total treatment duration in minutes (as a continuous variable). As shown in Table 2, none of the analyses revealed significant results. We also excluded outliers if indicated, however, the results remained non-significant.

### Relationship between effect size and quality criteria

In a series of subgroup analyses we examined the relationship between treatment efficacy and single quality criteria (see Table 3). Use of a treatment manual was a significant predictor of effect size. However, this analysis included four outliers and their exclusion produced non-significant results (*p* = 0.224). None of the other seven criteria was significantly associated with treatment efficacy. The examination of the relationship between study quality as a continuous variable including all eight items and treatment efficacy also produced non-significant results. Altogether, the findings are contrary to our hypothesis and indicate that high-quality trials produced similar treatment effects as lower-quality trials.

**Table 3.** Associations between quality criteria and effect sizes when compared to passive control conditions at posttreatment

	*k*	Hedges' *g*		*I*^2^	*p*
		*Trials not meeting criterion*	*Trials meeting criterion*		
Q1: Diagnosis	77	0.75	1.15	80.71***	0.074
(outlier-adjusted)	73	0.75	1.04	73.45***	0.125
Q2: Manual	77	1.68	1.04	80.84***	0.046*
(outlier-adjusted)	73	1.33	0.97	74.02***	0.224
Q3: Training	77	0.88	1.14	80.95***	0.193
(outlier-adjusted)	73	0.74	1.05	73.07***	0.075
Q4: Integrity	77	0.89	1.14	80.97***	0.196
(outlier-adjusted)	73	0.76	1.05	73.14***	0.078
Q5: ITT	77	1.10	1.08	81.54***	0.904
(outlier-adjusted)	73	0.98	1.00	74.27***	0.857
Q6: *N* ⩾ 50	77	1.21	0.94	80.99***	0.095
(outlier-adjusted)	73	1.05	0.94	74.07***	0.419
Q7: Randomization	77	1.20	1.02	81.13***	0.285
(outlier-adjusted)	73	1.07	0.95	73.89***	0.406
Q8: Blinding	77	1.14	1.08	81.47***	0.791
(outlier-adjusted)	73	0.98	1.00	74.20**	0.915
Quality sum score (outlier-adjusted)	*k*	*intercept*	*b*	*I^2^*	*p*
	77	1.15	−0.01	81.52***	0.837
	73	0.82	0.03	74.11***	0.520

ITT, analyses conducted on an intent-to-treat basis (as opposed to completer basis); *k*,  number of trials included in the analysis for the given comparison; *p* values refer to the comparison of trials meeting *v*. not meeting the quality criterion or the continuous moderator analyzed.

*p < 0.05, **p < 0.01, ***p < 0.001.

## Discussion

We aimed at providing a quantitative review of the efficacy of interventions for adults suffering from PTSD and at investigating the relationship between study quality and treatment efficacy. The results of 136 RCTs suggest that psychological interventions can effectively reduce PTSD symptoms. Our findings further indicate that study quality is not significantly associated with treatment efficacy.

Consistent with previous meta-analyses (e.g. Bisson et al., 2013; Cusack et al., 2016), our findings demonstrate the efficacy of psychological interventions for PTSD relative to PCC and ACC. This applies in particular to TF-CBT that produced medium-to-large effect sizes when compared to PCC and ACC at both posttreatment and follow-up. EMDR, too, produced medium-to-large effect sizes when compared to PCC and ACC. However, too few trials have examined the long-term efficacy of EMDR relative to control conditions. The comparison on TF-CBT produced nonsignificant effect sizes when compared to EMDR and when compared to other psychological treatments. Altogether, TF-CBT and EMDR have been most researched and appear to be effective at sustaining the reduction of symptoms of PTSD beyond treatment endpoint. These findings help inform which psychological interventions have strongest evidence of effect and should therefore be prioritized for clinical use when available. It is important to note, however, that 88% of the trials were conducted in Western countries, limiting the informative value for non-Western countries.

Our meta-analysis suggests that psychological interventions can produce large treatment effects irrespective of study quality. Contrary to findings from research on treatment for depression (Cuijpers et al., 2010), comparisons of high-quality trials with lower-quality trials produced only non-significant results as did the investigation of the single quality criteria. Two findings in particular strengthen the conclusion that psychological interventions for PTSD are efficacious. First, overall study quality was moderate with a mean of 5.82 on a scale from 0 to 8. Second, the finding that high-quality trials too produced large effect sizes further strengthens this conclusion. It remains unclear why our findings are different from those reported by Cuijpers et al. The comparison to the findings by Cuijpers et al. seems relevant as in both meta-analyses a similar number of trials was included (i.e. 136 in our meta-analysis *v.* 116 in Cuijpers et al.). Furthermore, the number of trials meeting all measured study quality criteria comprised about 10% of trials in both meta-analyses. One explanation may relate to overall study quality in each meta-analysis. In the current meta-analysis, 92% of the trials received at least half of the quality scores, which indicates that trial quality in general was not that poor. However, Cuijpers et al. only reported that 11 trials met all criteria and did not further report on the overall quality. Other relevant factors may be attributed to the heterogeneity in the diagnosis and overall effect sizes. Overall, depression is a much more heterogeneous disorder than PTSD and might be more difficult to treat. In fact, the overall treatment effects were larger in the current meta-analysis than in the one by Cuijpers et al. Our results are in line with a recent meta-analysis on the association between study quality and treatment efficacy for pediatric PTSD (Hoppen & Morina, 2020), which found that neither overall quality of the trials nor specific quality criteria were associated with effect sizes. Nonetheless, we must acknowledge that the number of high-quality trials is very small and therefore more high-quality trials need to be conducted to more rigorously examine treatment efficacy for PTSD. This applies in particular to other treatment forms than TF-CBT. Furthermore, many sub-analyses of long-term effects were limited by the low number of trials providing follow-up data. Accordingly, future research needs to investigate long-term effects of psychological interventions.

### Strengths and limitations

This work represents the first thorough examination of the association between study quality and treatment efficacy for PTSD. The inclusion of a total of 136 trials strengthens the validity of our findings. However, we also note some limitations. First, 45% of the trials reported treatment completer data and the remainder ITT data, which raises difficulties in interpretation of results. Second, some specific quality criteria, such as the criterion of having included at least 50 patients, may be criticized as rather arbitrary. Although we explicitly aimed at applying the criteria examined in relation to treatment of depression (Cuijpers et al., 2010), future research needs to use other quality measures. Recall, however, that the quality criteria that we applied here are based on the criteria for assessing the quality of treatment delivery originally recommended by Chambless and Hollon (1998) as well as the Cochrane Collaboration to assess trial quality (Higgins & Green, 2009). These two sets of criteria have played a decisive role in our understanding of what constitutes valid clinical trials that should inform empirically supported clinical work. Third, our ratings were based on the information reported in the specific publication, which may have resulted in rating some criterion as absent because the authors failed to report it. Finally, most of the included trials examined TF-CBT and the results mostly relate to this family of interventions.

## Conclusion

In sum, current published research indicates that trauma-focused treatments produce large treatment effects. This is in line with current treatment guidelines recommending trauma-focused treatments as first line interventions (Cusack et al., 2016). A substantial increase in the number of trials published in recent years resulted in a greater level of confidence in these findings, yet, more trials with follow-up assessments are needed. Treatment efficacy was not associated with study quality, which further supports the assumption that psychological interventions for PTSD are efficacious.

## Supplementary material

For supplementary material accompanying this paper visit https://doi.org/10.1017/S0033291721001641. click here to view supplementary material
