# Supplementary material for: Study quality and efficacy of psychological interventions for posttraumatic stress disorder: a meta-analysis of randomized controlled trials
Source: Psychol Med. 2021 May 12;51(8):1260–70. doi: 10.1017/S0033291721001641 (PMC8223238; doi:10.1017/S0033291721001641)
Supplement: Supplementary file 1 [file S0033291721001641sup001.docx]

**Manuscript: Study quality and efficacy of psychological interventions for posttraumatic stress disorder: A meta-analysis of randomized controlled trials**

**Appendix A. Trial Characteristics**

**Appendix B. Trial Quality**

**Appendix C. References of the included studies**

**Appendix D. Number of active and control conditions examined in the included studies**

**Appendix E. Efficacy of psychological interventions including outlier- and asymmetry-adjustments**

**Appendix F. Comparison of high quality (HQ) and lower quality trials (Other) when compared to passive control conditions at posttreatment including outlier- and asymmetry-adjustments**

| Appendix A. Trial Characteristics | | | | | | | | | | | |
| --- | --- | --- | --- | --- | --- | --- | --- | --- | --- | --- | --- |
| Publication, conditions,  (number & length of sessions) | N at post-treatment | % PTSD diagnosis baseline | Outcome measure | Country | Mean age (SD or range) | Longest follow-up in months | Treatment format | Stat. analysis | % female | Type of trauma | Study quality |
| **Acarturk et al., 2016^1^**  **EMDR (7 sessions, 90 min.)**  **WL** | 49  49 | 100 | IES-R | Turkey (Syrian refugees) | 33.32 (11.09)  34.04 (10.00) | 1 | Individual | ITT | 74 | Mass conflict | 8 |
| **Akbarian et al., 2015^2^**  **CBT (10 sessions, 60 – 90 min.)**  **TAU (n.r.)** | 14  14 | 100 | IES-R | Iran | 32.07 (5.76)  31.21 (6.10) | n.a. | Group  Individual | ITT | 79 | Multiple types | 5 |
| **Asukai et al., 2010^3^**  **PE (8-15 sessions, 90 min.)**  **TAU (n.r.)** | 12  12 | 100 | CAPS | Japan | 27.10 (5.40)  31.40 (8.80) | r.b.i. | Individual  Individual | ITT | 88 | Multiple types | 7 |
| **Basoglu et al., 2005^4^**  **SSBT (1 session, 60 min.)**  **WL** | 31  28 | 100 | CAPS | Turkey | 36.30 (11.50) | r.b.i. | Individual | Compl. | 84 | Disaster | 6 |
| **Basoglu et al., 2007^5^**  **CBT (1 session, 69 – 130 min.)**  **WL** | 16  15 | 100 | CAPS | Turkey | 34.00 (11.00) | r.b.i. | Individual | Compl. | 87 | Disaster | 6 |
| **Beck et al., 2009^6^**  **CBT (14 sessions, 120 min.)**  **MA** | 17  16 | 100 | CAPS | USA | 43.30 (12.80) | r.b.i. | Group  Individual | Compl. | 82 | Motor vehicle accident | 5 |
| **Belleville et al., 2018^7^**  **IRT (5 sessions, 60 min.)**  **WL** | 19  20 | 100 | MPSS | Canada | 29.45 (9.05)  31.45 (10.32) | r.b.i. | Individual | ITT | 88 | Sexual assault | 6 |
| **Bisson et al., 2020^8^**  **3MDR (9 sessions, average 63.3 min)**  **WL** | 16  19 | 100 | CAPS | UK | 40.2 (10.13)  44.0 (11.97) | r.b.i. | Individual | Compl. | 0 | Multiple types | 6 |
| **Blanchard et al., 2003^9^**  **CBT (10 sessions, n.r.)**  **SPT (10 sessions, n.r.)**  **WL** | 37  36  25 | 77.78  77.78  87.50 | CAPS | USA | 40.60 (11.00)  40.60 (13.10)  42.10 (10.90) | 3 | Individual  Individual | ITT | 29  29  60 | Motor vehicle accident | 6 |
| **Bohus et al., 2013^10^  DBT-PTSD (23 sessions, 45 min.)   TAU (n.r.)** | 36  38 | 100 | CAPS | Germany | 35.14 (10.60)  36.71 (9.84) | 6 | Individual  Individual | ITT | 100 | Childhood sexual abuse | 7 |
| **Bohus et al., 2020^11^  CPT (45 sessions, 50 min.)  DBT-PTSD (45 sessions, 50 min.)** | 95  98 | 100 | CAPS | Germany | 35.5 (11.4)  37.0 (10.7) | n.a. | Individual  Individual | ITT | 100 | Childhood sexual and/or physical abuse | 8 |
| **Bormann et al., 2008^12^  Mantram interv. (6 sessions, 90 min.)  WL** | 14  15 | 100 | CAPS | USA | 56.00 (6.57) | n.r. | Group | Compl. | 0 | Combat | 5 |
| **Bormann et al., 2013^13^  Mantram interv. (6 sessions, 90 min.)  TAU (n.r.)** | 71  75 | 100 | CAPS | USA | 57.00 (10.10) | r.b.i. | Group  Individual | ITT | 3 | Combat | 6 |
| **Boterhoven de Haan et al., 2020^14^  EMDR (12 sessions, 90 min.)  IR (12 sessions, 90 min.)** | 68  66 | 93 | CAPS | Australia  Germany  NL | 38.96 (11.51)  38.08 (10.85) | 12 | Individual  Individual | ITT | 80  73 | Multiple types during childhood | 7 |
| **Brom et al., 2017^15^   SE (15 sessions, 60 min.)   WL** | 32  28 | 100 | CAPS | Israel | 40.51 (13.05) | r.b.i. | Individual  Individual | Compl. | 46  57 | Multiple types | 7 |
| **Bryant et al., 2003^16^**  **IE (8 sessions, 90 min.)   SC (8 sessions, 90 min.)** | 20  18 | 100 | CAPS | Australia | 37.05 (12.31)  36.28 (8.41) | 6 | Individual  Individual | ITT | 52  52 | Nonsexual assault or motor vehicle accident | 6 |
| **Bryant et al., 2011^17^   CBT (8 sessions, 60 min.)   TAU (8 sessions, n.r.)** | 16  12 | 100 | PSS-I | Thailand | 42.30 (6.30)  43.9 (11.9) | 3 | Individual  Individual | ITT | 100  91 | Terror | 5 |
| **Bryant et al., 2019^18^  CBT (12 sessions, 90 min.)**  **WL** | 33  34 | 100 | CAPS | Australia | 44.7 (10.7)  43.4 (7.8) | r.b.i. | Individual | Compl. | 12  19 | Multiple types | 4 |
| **Butollo et al., 2016^19^  DET (24 sessions, n.r.)   CPT (24 sessions, n.r.)** | 74  67 | 100 | PDS | Germany | 37.99 (12.10)  33.67 (10.30) | 6 | Individual  Individual | ITT | 65  68 | Multiple types | 7 |
| **Capezzani et al., 2013^20^  EMDR (8 sessions, n.r.)   CBT (8 sessions, n.r.)** | 21  11 | 100 | IES-R | Italy | 52.70 (8.68)  50.82 (7.64) | n.a. | Individual  Individual | ITT | 100 | Cancer | 4 |
| **Carletto et al., 2016^21^  EMDR (10 sessions, 60 min.)**  **RT (n.r.)** | 20  22 | 100 | CAPS | Italy | 39.52 (11.68)  40.66 (10.03) | n.a. | Individual | Compl. | 75  86 | MS | 6 |
| **Carlson et al., 1998^22^  EMDR (12 sessions, 60-75 min.)   TAU (n.r.)** | 10  12 | 100 | M-PTSD | USA | 52.70 (8.60)  46.90 (4.00) | r.b.i. | Individual  Individual | Compl. | 0  0 | Combat | 4 |
| **Carlsson et al., 2018^23^  CR (16 sessions, 45-60 min.)  SIT (16 sessions, 45-60 min.)** | 64  62 | 100 | HTQ | Denmark | 43.30 (9.50) | n.a. | Individual | ITT | 44 | Multiple types | 7 |
| **Carter et al., 2013^24^**  **SKY (5 sessions, 22 hours total)  WL** | 14  11 | 100 | CAPS | Australia | 58.50 (3.80)  58.40 (4.80) | r.b.i. | Group | Compl. | 0 | Combat | 5 |
| **Castillo et al., 2016^25^  CBT (16 sessions, 90 min.)   WL** | 42  42 | 100 | CAPS | USA | 35.90 (11.00) | r.b.i. | Group  Individual | ITT | 100 | Multiple types | 6 |
| **Chard et al., 2005^26^  CPT (17 sessions à 90 min. group +   10 sessions à 60 min. individual)   MA (17 phone-calls à 5-10 min.)** | 28  27 | 100 | CAPS | USA | 32.77 (8.87) | r.b.i. | Combination  Individual | Compl. | 100 | Childhood sexual abuse | 6 |
| **Classen et al., 2020^27^  TBG (20 sessions, n.r.)  WL** | 14  18 | 100 | PCL | Canada | 43.51 (10.01) | 6 | Group | Compl. | 100 | Childhood sexual and/or physical abuse | 1 |
| **Cloitre et al., 2002^28^   STAIR (16 sessions, 8 à 60 min. + 8  à 90 min.)  WL** | 31  27 | 100 | CAPS | USA | 34.99 (7.22) | r.b.i. | Individual  Individual | ITT | 100 | Childhood sexual and/or physical abuse | 7 |
| **Cloitre et al., 2010^29^  STAIR (16 sessions, 90 min.)  Skills comparator (n.r.)** | 33  38 | 100 | CAPS | USA | 33.20 (n.r.)  37.10 (n.r.) | 6 | Individual  Individual | ITT | 100 | Childhood sexual and/or physical abuse | 8 |
| **Cottraux et al., 2008^30^**  **CBT (10-16 sessions, 60-120 min.)  ST (16 sessions, 60 min.)** | 27  15 | 100 | PCL | France | 43.18 (10.60)  37.20 (9.20) | r.b.i. | Individual  Individual | Compl. | 70 | Multiple types | 6 |
| **Davis et al., 2020^31^  HYP (16 sessions, 90 min.)  WLP (16 sessions, 90 min.)** | 70  70 | 100 | CAPS | USA | 49.9 (12.6)  51.2 (13.3) | 7 | Group  Group | Compl. | 34 | Multiple types | 6 |
| **Devilly et al., 1998^32^  EMDR (2 sessions, 90 min.)  TAU (n.r.)** | 12  10 | 100 | M-PTSD | Australia | 50.10 (6.48) | r.b.i. | Individual n.r. | Compl. | 0 | Combat | 3 |
| **Devilly et al., 1999^33^  EMDR (8 sessions, 90-165 min.)  TTP (9 sessions, 90-165 min.)** | 11  12 | 100 | PTSD-I | Australia | 37.96 (12.82) | r.b.i. | Individual  Individual | Compl. | 93  58 | Multiple types | 4 |
| **Dorrepaal et al., 2012^34^  TF-CBT (20 sessions, 120 min.)  TAU (n.r.)** | 38  33 | 100 | DTS | NL | 40.30 (10.70)  37.10 (10.30) | n.a. | Group  Individual | ITT | n.r. | Childhood sexual and/or physical abuse | 6 |
| **Duffy et al., 2007^35^  CBT (12 sessions, n.r.)   WL** | 29  29 | 100 | PDS | Northern Ireland | 44.1 (11.3)  43.7 (12.3) | r.b.i. | Individual  Individual | ITT | 34  45 | Terror | 7 |
| **Dunne et al., 2012^36^  CBT (10 sessions, 60 min.)  WL** | 13  13 | 100 | PDS | Australia | 32.54 (7.09) | r.b.i. | Individual  Individual | ITT | 50  50 | Chronic whiplash | 4 |
| **Echeburua et al., 1997^37^  EXP+CR (6 sessions, 7h total)   PMR (6 sessions, 4,15h total)** | 10  10 | 100 | Global Scale of PTSD | Spain | 20.00 (7.09) | 12 | Individual  Individual | ITT | 100 | Sexual assault | 4 |
| **Ehlers et al., 2003^38^  CBT (12 sessions, 60-90min.)  Self-help booklet (1 session, 40min.)   WL** | 28  28  29 | 100 | PDS | UK | n.r. | 6 | Individual  Individual  Individual | ITT | n.r.  n.r.  n.r. | Motor vehicle accident | 7 |
| **Ehlers et al., 2005^39^  CT (12 sessions, 60-90 min.)   WL** | 14  14 | 100 | PDS | UK | 35.4 (10.9)  37.8 (11.2) | r.b.i. | Individual  Individual | ITT | 57  50 | Multiple types | 6 |
| **Ehlers et al., 2014^40^   CT (12 sessions, up to 20h total)   EFST (12 sessions, up to 20h total)  WL** | 31  30  30 | 100 | CAPS | UK | 42.50 (11.70)  37.80 (9.90)  36.80 (10.50) | 10 | Individual  Individual  Individual | ITT | 58  57  60 | Multiple types | 8 |
| **Engel et al., 2015^41^**  **DESTRESS-PC (18 sessions 15-30  min. + 30 min. homework)   TAU (3 sessions, 15 min.)** | 29  29 | 100 | PCL | USA | 36.20 (7.75)  36.70 (9.75) | 4 | Individual | Compl. | 21  16 | Combat and military sexual trauma | 7 |
| **Fecteau et al., 1999^42^  CBT (4 sessions, 90-180 min.)   WL** | 10  10 | 100 | CAPS | Canada | 41.30 (25–63) | r.b.i. | Individual  Individual | Compl. | 70  70 | Motor vehicle accident | 6 |
| **Foa et al., 1991^43^   PE (9 sessions, 90 min.)   SIT (9 sessions, 90 min.)**   **SC (9 sessions, 90 min.)  WL** | 10  14  11  10 | 100 | SI-PTSD | USA | 32.70 (7.30)  29.30 (6.30)  34.20 (9.80)  32.00 (9.60) | r.b.i. | Individual  Individual  Individual | Compl. | 100 | Sexual assault | 5 |
| **Foa e al., 1999^44^   PE (9 sessions, 2x120+7x90 min.)  SIT (9 sessions, 2x120+7x90 min.)  WL** | 23  19  15 | 100 | PSS-I | USA | 34.90 (10.60) | 12 | Individual  Individual  Individual | Compl. | 100 | Sexual or non-sexual assault | 5 |
| **Foa et al., 2005^45^  PE (12 sessions, 90-120 min.)   WL** | 52  25 | 100 | PSS-I | USA | 31.30 (9.89) | r.b.i. | Individual  Individual | ITT | 100 | Sexual or non-sexual assault | 7 |
| **Foa et al., 2018^46^  PE (10 sessions, 90 min.)**  **PCT (10 sessions, 90 min.)**  **MA (4 sessions, 10-15 min.)** | 109  107  40 | 100 | PSS-I | USA | 32.89 (7.05)  32.54 (7.45)  32.70 (7.68) | 6 | Individual  Individual  Individual | ITT | 9  15  5 | Combat | 8 |
| **Forbes et al., 2012^47^  CPT (12 sessions, 60 min.)**  **TAU (n.r.)** | 30  29 | 100 | CAPS | Australia | 53.13 (13.97)  53.62 (13.33) | 3 | Individual  Individual | ITT | 7  0 | Combat | 7 |
| **Ford et al., 2011^48^  TARGET (12 sessions, 50 min.)   PCT (12 sessions, n.r.)   WL** | 48  53  45 | 80  74  87 | CAPS | USA | 30.70 (6.90) | r.b.i. | Individual  Individual  Individual | ITT | 100  100  100 | Multiple types | 6 |
| **Ford et al., 2013^49^  TARGET (12 sessions, 75 min.)   SGT (12 sessions, 75 min.)** | 38  34 | 82  74 | CAPS | USA | 34.60 (8.60)  38.00 (7.80) | n.a. | Individual  Group | Compl. | 100  100 | Multiple types | 6 |
| **Galovski et al., 2012^50^  MCPT (4-18 sessions, n.r.)  SMDT (n.r.)** | 38  37 | 100 | CAPS | USA | 39.80 (11.74) | r.b.i. | Individual | ITT | 69 | Sexual or physical assault | 8 |
| **Gersons et al., 2000^51^  BEP (16 sessions, 60 min.)  WL** | 22  20 | 100 | SI-PTSD | NL | 35.00 (6.00)  38.00 (7.00) | 3 | Individual | ITT | 18  5 | Multiple types | 7 |
| **Ghafoori et al., 2017^52^  PE (12 sessions, 60-90 min.)  PCT (12 sessions, 60-90 min.)** | 47  24 | 100 | PCL-5 | USA | 35.10 (12.80)  35.30 (10.40) | n.a. | Individual  Individual | ITT | 83  83 | Physical assault and other types | 6 |
| **Goldstein et al., 2017^53^   IN EXC   (36 sessions, 60 min.)   WL** | 21  26 | 89.36 | CAPS | USA | 46.80 (14.93) | n.a. | Group  Individual | ITT | 19 | Combat | 4 |
| **Gray et al., 2019^54^  RTM (3 sessions, 120 min.)  WL** | 37  37 | 100 | PSS-I | USA | 48.60 (13.30) | r.b.i. | Individual | ITT | 0 | Combat and other types | 7 |
| **Hensel-Dittmann et al., 2011^55^  NET (10 sessions, 90 min.)  SIT (10 sessions, 90 min.)** | 11  10 | 100 | CAPS | Germany | n.r. | 6 | Individual  Individual | Compl. | n.r. | Multiple types | 6 |
| **Hinton et al., 2005^56^  CBT (12 sessions, n.r.)  TAU (n.r.)** | 20  20 | 100 | CAPS | USA (Cambod. refugees) | 50.90 (6.11)  52.70 (7.43) | r.b.i. | Individual | ITT | 60  60 | Multiple types | 7 |
| **Hinton et al., 2011^57^  CBT (14 sessions, 60 min.)  AMR (14 sessions, 60 min.)** | 12  12 | 100 | PCL | USA (Cambod. refugees) | 47.60 (8.20)  51.40 (5.90) | 3 | Group  Group | ITT | 100 | n.r. | 5 |
| **Hollifield et al., 2007^58^  CBT (12 sessions, 120 min.)   WL** | 25  24 | 100 | PSS-SR | USA | 40.90 (13.40) 43.40 (13.50) | 3 | Group | ITT | 79  63 | Multiple types | 5 |
| **Ivarsson et al., 2014^59^  I-CBT (8 sessions, 28 min. of contact to therapists on average)**  **SC (n.r.)** | 31  31 | 100 | IES-R | Sweden | 44.80 (11.20)  47.20 (12.20) | r.b.i. | Individual | ITT | 77  87 | Multiple types | 8 |
| **Jacob et al., 2014^60^  NET (8 sessions, 90-150 min.)**  **WL** | 38 (17 orphans)  38 (16 orphans) | 100 | CAPS | Rwanda | widows 48.29 (13.40) orphans 25.06 (4.31)  widows  46.86 (11.73) orphans 24.00 (4.40) | r.b.i. | Individual | ITT | 89  95 | Multiple types | 8 |
| **Jalal et al., 2020^61^  CA-CBT (14 sessions, 60 min.)  AMR (14 sessions, 60 min.)** | 10 10 | 100 | PCL-C | South Africa | 28.2 (15 - n.r.) | n.a. | Individual | Compl. | 75 | Multiple types | 3 |
| **Jensen, 1994^62^  EMDR (3 sessions, n.r.)  TAU (n.r.)** | 13  12 | 100 | SI-PTSD | USA | 43.10 (2.84) | n.a. | Individual  Individual | Compl. | 0  0 | Combat | 3 |
| **Johnson et al., 2011^63^  HOPE (12 sessions, 60-90 min.)**  **TAU (n.r.)** | 34  34 | 88.60  85.70 | CAPS | USA | 32.55 (8.00) | 6 | Individual  Group | Compl. | 100 | IPV | 5 |
| **Johnson et al., 2016^64^**  **HOPE (16 sessions, 60 min.)**  **TAU (n.r.)** | 26  25 | 93.30  96.70 | CAPS | USA | 33.30 (10.48) 33.20 (10.39) | 6 | Individual  Group | Compl. | 100 | IPV and other types | 6 (Post + FU1) 5 (FU2) |
| **Keane et al., 1989^65^  IT (14 sessions, 90 min.)   WL** | 11  13 | 100 | MMPI  PTSD | USA | 34.70 (4.30)  34.50 (2.10) | r.b.i. | Individual | Compl. | 0  0 | Combat | 2 |
| **Kent et al., 2011^66^  ROT (12 sessions, 90 min.)  WL** | 23  19 | 100 | PDS | USA | 54.00 (8.34) | n.a. | Group | ITT | 33 | Multiple types | 4 |
| **Krakow et al., 2000^67^  IRT (3 sessions, 2x 180+1x 60 min.)   WL** | 42  48 | 95  95 | PSS-SR | USA | Compl.  40.10 (11.30)  36.00 (9.80) | r.b.i. | Group | Compl. | 100 | Sexual assault and other types | 3 |
| **Krakow et al., 2001^68^**  **IRT (3 sessions, 2x 180+1x 60 min.)   WL** | 45  52 | 95  95 | CAPS | USA | Compl.  40.00 (11.20)  36.00 (9.30) | r.b.i. | Group  Individual | Compl. | 100 | Sexual assault and other types | 4 |
| **Krupnick et al., 2008^69^  IPT (16 sessions, 120 min.)  WL** | 26^a^  10^a^ | 100 | CAPS | USA | 32.00 (10.20) | 4 | Group | ITT | 100 | Multiple types | 5 |
| **Kubany et al., 2003^70^**  **CTT-BW (8-11 sessions, 90 min.)  WL** | 19  18 | 100 | CAPS | USA | Compl.  36.80 (9.50) | r.b.i. | Individual | ITT | 100 | IPV and other types | 5 |
| **Kubany et al., 2004^71^  CTT-BW (8-11 sessions, 90 min.)   WL** | 63  62 | 100 | CAPS | USA | 42.20 (10.10) | r.b.i. | Individual  Individual | ITT | 100  100 | IPV and other types | 7 |
| **Lang et al., 2019^72^  CM (10 sessions, 90 min.)  VC (10 sessions, 90 min.)** | 14  14 | 100 | CAPS | USA | 49.10 (14.50) | n.a. | Group  Group | Compl. | 25 | Combat | 5 |
| **Langkaas et al., 2017^73^**  **PE (10 sessions, 90-120 min.)  IR (10 sessions, 90-120 min.)** | 34  31 | 100 | PSS-I | Norway | 45.20 (9.70) | 12 | Individual  Individual | ITT | 58 | Multiple types | 8 |
| **Laugharne et al., 2016^74^  PE (12 sessions, n.r.)  EMDR (12 sessions, n.r.)** | 10  10 | 100 | CAPS | Australia | 40.10 (9.90) | n.a. | Individual  Individual | ITT | 70 | Multiple types | 7 |
| **Lee et al., 2002^75^  EMDR (7 sessions, 90 min.)   PE + SIT (7 sessions, 90 min.)** | 12  12 | 100 | SI-PTSD | Australia | 35.30 (n.r.) | 3 | Individual  Individual | Compl. | 46  46 | Multiple types | 4 |
| **Lely et al., 2019^76^  NET (11 sessions, 90 min.)  PCT (11 sessions, 90 min.)** | 18  15 | 100 | CAPS | NL | 62.65 (5.89)  62.47 (6.24) | 4 | Individual  Individual | ITT | 28  27 | Multiple types | 7 |
| **Lewis et al., 2017^77^  I-CBT (n.r.)  WL** | 21  21 | 100 | CAPS | UK | 39.29 (12.70) | 3 | Individual | ITT | 60 | Multiple types | 7 |
| **Lindauer et al., 2005^78^  BEP (16 sessions, 45-60 min.)  WL** | 12  12 | 100 | SI-PTSD | NL | 37.16 (10.20)  40.30 (8.90) | n.a. | Individual | ITT | 42  67 | Multiple types | 7 |
| **Littleton et al., 2016^79^  FSTTP (n.r.)  PESHW (n.r.)** | 23 28 | 100 | PSS-I | USA | 22 (18-42) | 3 | Individual Individual | Compl. | 100 | Rape and other IPV | 6 (Post) 5 (FU1) |
| **Litz et al., 2007^80^  I-CBT (n.r.)  I-SC (n.r.)** | 14  17 | 100 | PSS-I | USA | 39.86 (7.72)  38.63 (9.41) | 6 | Individual | Compl. | 19  25 | Combat or terror | 5 |
| **Maguen et al., 2017^81^  IOK (6-8 sessions, 60-90min.)  TAU (n.r.)** | 15  15 | 100 | PCL-M | USA | 61.20 (13.00) | n.a. | Individual  Individual | Compl. | 0  0 | Combat | 4 |
| **Marcus et al., 1997^82^  EMDR (8 sessions, 50 min.)  TAU (n.r., individual therapy: 50  min., group therapy: 90 min.)** | 33  33 | 100 | MPSS | USA | Women 39.98 (18.00-73.00)  Men 44.78 (23.00-67.00) | n.a. | Individual  Combination | Compl. | 79  79 | Multiple types | 5 |
| **Markowitz et al., 2015^83^  PE (10 sessions, 90 min.)  IPT (14 sessions, 50 min.)   RT (10 sessions, 90 min.)** | 38  40  32 | 100 | CAPS | USA | 47.50 (10.60)  41.00 (9.10)  34.80 (5.10) | 3 | Individual  Individual  Individual | ITT | 55  70  88 | Multiple types | 6 |
| **Marks et al., 1998^84^  EXP (10 sessions, 90 min.)  EXP + CR (10 sessions, 105 min.)   RT (10 sessions, 90 min.)** | 20  18  20 | 100 | CAPS | UK | 39.00 (11.00)  38.00 (9.00)  36.00 (10.00) | 3 | Individual  Individual  Individual | Compl. | 39  31  48 | Multiple types | 4 |
| **McDonagh et al., 2005^85^  CBT (14 sessions, 7x120+7x90 min.)  PCT (14 sessions, 7x120+7x90 min.)**  **WL** | 29  22  23 | 100 | CAPS | USA | 39.80 (9.90)  39.60 (9.60)  42.00 (9.80) | 6 | Individual  Individual | ITT | 100 | Childhood sexual abuse | 6 |
| **Mitchell et al., 2014^86^  YI (12 sessions, 75 min.)   WL** | 20  18 | 71  71 | PCL | USA | 44.37 (12.37) | 1 | Group  Individual | ITT | 100 | Multiple types | 5 |
| **Monson et al., 2006^87^  CPT (12 sessions, n.r.)  WL** | 30  30 | 100 | CAPS | USA | 54.00 (6.30) | 1 | Individual | ITT | 10 | Combat | 7 |
| **Monson et al., 2012^88^  CBCT (15 sessions, n.r.)  WL** | 20  20 | 100 | CAPS | USA & Canada | 40.40 (11.30)  33.80 (10.50) | r.b.i. | Couple | ITT | 65  85 | Multiple types | 7 |
| **Morath et al., 2014^89^  NET (12 sessions, n.r.)   WL** | 19  19 | 100 | CAPS | Germany | 28.70 (9.54)  30.10 (8.21) | r.b.i. | Individual  Individual | Compl. | 32  32 | Multiple types | 6 |
| **Mueser et al., 2008^90^  CBT (16 sessions, n.r.)  TAU (n.r.)** | 32  27 | 100 | CAPS | USA | 45.13 (9.83)  43.30 (11.41) | 6 | Individual  Individual | Compl. | 76  82 | Multiple types | 5 |
| **Nacasch et al., 2011^91^  PE (9-15 sessions, 90-120 min.)  TAU (n.r., 60 min.)** | 15  15 | 100 | PSS-I | Israel | 34.80 (11.40)  33.70 (11.90) | 12 | Individual  Individual | ITT | n.r. | Combat and terror | 7 |
| **Neuner et al., 2004^92^  NET (4 sessions, 90-120 min.)   SC (4 sessions, 90-120 min.)   PSY-EDU (1 session, n.r.)** | 17  14  12 | 100 | PDS | Uganda (Sundanese refugees) | 31.90 (6.70)  33.80 (7.90)  34.20 (6.90) | 12 | Individual  Individual  Individual | Compl. | 53  57  75 | Multiple types | 6 |
| **Neuner et al., 2008^93^  NET (6 sessions, n.r.)   TC (6 sessions, n.r.)   WL** | 111  111  55 | 100 | PDS | Uganda (Rwandan & Somal. refugees) | 34.40 (12.20)  35.20 (12.80)  35.60 (14.00) | 6 | Individual  Individual  Individual | ITT | 51  53  49 | Multiple types | 7 |
| **Neuner et al., 2010^94^  NET (5-17 sessions, 120 min.)  TAU (n.r.)** | 14^b^  16 | 100 | PDS | Germany (refugees) | 31.10 (7.80)  31.60 (7.70) | 6 | Individual Individual | ITT | 31  31 | Physical torture and other types | 7 |
| **Nidich et al., 2018^95^  PE (12 sessions, 90 min.)  TMed (12 sessions, 90 min.)  HE (12 sessions, 90 min.)** | 68  68  66 | 100 | CAPS | USA | 48.50 (15.60)  46.40 (14.30)  46.20 (16.40) | n.a. | Individual  Individual  Individual | ITT | 18  18  15 | Combat | 7 |
| **Nijdam et al., 2012^96^  EMDR (17 sessions, 90 min.)  BEP (16 sessions, 45-60 min.)** | 70  70 | 100 | SI-PTSD | NL | 38.30 (12.20)  37.30 (10.60) | n.a. | Individual  Individual | Compl. | 51  61 | Assault and other types | 7 |
| **Niles et al., 2012^97^  MM (8 sessions; 2 f2f à 45 min., 6 tele à 20 min.)  PSY-EDU (8 sessions; 2 f2f à 45 min., 6 tele à 20 min.)** | 13  14 | 100 | CAPS (post) PCL (FU) | USA | 52.00 (13.00) | 1.50 | Individual  Individual | Compl. | 0 | Combat or mass violence (as peacekeepers) | 5 |
| **Orang et al., 2018^98^  NET (12 sessions, 120-150 min.)  TAU (12 sessions, 90–120 min.)** | 17^b^  17^b^ | 100 | PSS-I | Iran | 38.04 (9.69)  37.28 (7.92) | 6 | Individual Combinat. | Compl. | 100 | IPV | 5 |
| **Pacella et al., 2012^99^  PE (10 sessions, 90-120 min.)  WL** | 34  24 | 100 | PSS-I | USA | 46.00 (5.80)  48.00 (7.00) | 3 | Individual | ITT | 37 | HIV-related and others | 8 |
| **Paunovic, 2011^100^  EIT (3-9 sessions, 60-120 min.)   WL** | 14  15 | 100 | CAPS | Sweden | 37.10 (13.80)  37.30 (10.20) | r.b.i. | Individual  Individual | Compl. | 50  47 | Sexual assault and other types | 2 |
| **Power et al., 2002^101^**  **EXP+CR (10 sessions, 90 min.)  EMDR (10 sessions, 90 min.)  WL** | 21  27  24 | 100 | SI-PTSD | UK | 43.20 (11.00)  38.60 (11.80)  36.50 (11.60) | r.b.i. | Individual  Individual | Compl. | 44  38  42 | Multiple types | 6 (EMDR vs WL)  5 (other comparisons) |
| **Rauch et al., 2015^102^  PE (10-12 sessions, 80 min.)   PCT (10-12 sessions, 80 min.)** | 11  15 | 100 | CAPS | USA | 31.90 (7.60) | n.a. | Individual  Individual | Compl. | 8  8 | Combat | 4 |
| **Ready et al., 2018^103^  GBET (32 sessions, 240 min.)  GPCT (37 sessions, 90 min.)** | 40  41 | 100 | CAPS | USA | 61.40 (2.60) | r.b.i. | Group  Group | Compl. | n.r. | Combat | 5 |
| **Reger et al., 2016^104^  VRET (10 sessions, 90-120 min.)  PE (10 sessions, 90-120 min.)  WL** | 54  54  54 | 100 | CAPS | USA | 29.52 (6.47)  30.89 (7.09)  30.39 (6.45) | r.b.i. | Individual  Individual  Individual | ITT | 6  4  2 | Nonsexual  assault | 8 |
| **Resick et al., 2002^105^  CPT (12 sessions, 13 hours total)  PE (9 sessions, 13 hours total)   WL** | 62  62  47 | 100 | SI-PTSD | USA | 32.00 (9.90) | 9 | Individual  Individual  Individual | ITT | 100  100  100 | Sexual assault | 6 |
| **Resick et al., 2015^106^  GCPT (12 sessions, 90 min.)  GPCT (12 sessions, 90 min.)** | 56  52 | 100 | PSS-I | USA | 31.80 (7.30)  32.40 (7.90) | 12 | Group  Group | ITT | 7  8 | Combat and other types | 6 |
| **Robjant et al., 2019^107^  NET (6 sessions á 90-120 min.   individual, 6 sessions á 90-120 group)  TAU (n.r.)** | 45 43 | 100 | PSS-I | DRC | 18 (16-25)  18 (16-25) | 9 | Combination  Individual | Compl. | 100 | Child soldiers | 7 |
| **Rothbaum et al., 2005^108^  EMDR (9 sessions, 90 min.)   PE (9 sessions, 90 min.)   WL** | 20  20  20 | 100 | CAPS | USA | 33.80 (11.00) | 6 | Individual  Individual  Individual | Compl. | 100 | Sexual assault | 5 |
| **Sautter et al., 2015^109^  SAT (12 sessions, 60 min.)  PFE (12 sessions, 60 min.)** | 29  28 | 100 | CAPS | USA | 32.55 (6.16)  33.71 (7.01) | 3 | Couple  Couple | ITT | 0  3.6 | Combat | 7 |
| **Schaal et al., 2009^110^  NET (4 sessions, 120-150 min.)  IPT (4 sessions, 120-150 min.)** | 12 14 | 100 | CAPS | Rwanda | 19.42 (3.59) | 6 | Individual  Group | ITT | 38.46 | Mass Conflict | 7 |
| **Scheck et al., 1998^111^  EMDR (2 sessions, 90 min.)  AL (2 sessions, 90 min.)** | 28  29 | 77 | IES | USA | 20.93  (16.00-25.00) | r.b.i. | Individual  Individual | Compl. | 100 | Multiple Types | 5 |
| **Schnurr et al., 2003^112^  TFGT (30 sessions, 90-120 min.)  GPCT (30 sessions, 90-120 min.)** | 162  160 | 100 | CAPS | USA | 50.60 (3.70)  50.80 (3.80) | 5 | Group  Group | Compl. | 0 | Combat | 6 |
| **Schnurr et al., 2007^113^  PE (10 sessions, 90 min.)  PCT (10 sessions, 90 min.)** | 141 143 | 100 | CAPS | USA | 44.60 (9.39)  44.90 (9.47) | 6 | Individual  Individual | ITT | 100 | Sexual Trauma and other types | 7 |
| **Sloan et al., 2012^114^  WET (5 sessions, 1x60+4x40 min.)   WL** | 22  24 | 100 | CAPS | USA | 40.65  (13.10) | 4.5 | Individual  Individual | ITT | 65  65 | Motor Vehicle Accident | 6 |
| **Sloan et al., 2018^115^  GCBT (14 sessions, 120 min.)  GPCT (14 sessions, 120 min.)** | 98  100 | 100 | CAPS | USA | 54.40 (11.44)  57.22 (12.51) | 12 | Group  Group | ITT | 0 | Combat and other Types | 8 |
| **Stanbury et al., 2020^116^  PE (12 sessions, 90 min.)**  **EMDR (12 sessions, 90 min.)** | 10 10 | 100 | CAPS | Australia | 44.60 (12.18) 39.70 (9.55) | r.b.i. | Individual Individual | Compl. | n.r. | Multiple types | 5 |
| **Stenmark et al., 2013^117^  NET (10 sessions, 90 min.)  TAU (10 sessions, 90 min.)** | 33  21 | 100 | CAPS | Norway  (refugees) | 34.50 (11.10)  36.60 (11.00) | 6 | Individual  Individual | Compl. | 33  27 | Multiple Types | 7 |
| **Suris et al., 2013^118^   CPT (12 sessions, n.r.)  PCT (12 sessions, n.r.)** | 52  34 | 100 | CAPS | USA | 44.60 (10.50)  48.40 (8.20) | 12 | Individual  Individual | Compl. | 83  88 | Military Sexual Trauma | 7 |
| **Taylor et al., 2003^119^**  **PE (8 sessions, 90 min.)**  **EMDR (8 sessions, 90 min.)**  **RT (8 sessions, 90 min.)** | 15  15  15 | 100 | CAPS | Canada | 37.00 (10.00) | 3 | Individual  Individual  Individual | ITT | 75 | Multiple types | 6 |
| **ter Heide et al., 2016^120^**  **EMDR (9 sessions, 3x60+6x90 min.) TAU (12 sessions, 60 min.)** | 32  29 | 100 | CAPS | NL | 43.10 (10.70)  39.80 (11.90) | 3 | Individual  Individual | Compl | 17  39 | Multiple types | 7 |
| **Thorp et al., 2019^121^**  **PE (12 sessions, 90 min.)**  **RT (12 sessions, 90 min.)** | 29  38 | 100 | CAPS | USA | 66.51 (6.21)  64.43 (4.49) | 6 | Individual  Individual | ITT | 0 | Combat | 6 (Post) 5 (FU2) |
| **Tylee et al., 2017^122^  RTM (3 sessions, 120 min.)  WL** | 15 15 | 100 | PSS-I | USA | 49.0 (19.5) 42.6 (15.9) | r.b.i. | Individual | Compl. | 0 | Combat and other types | 5 |
| **van den Berg et al., 2015^123^**  **EMDR (8 sessions, 90 min.)**  **PE (8 sessions, 90 min.)**  **WL** | 55  55  47 | 100 | CAPS | NL | 40.40 (11.30)  42.60 (10.30)  40.30 (9.70) | 6 | Individual  Individual | ITT | 55  57  51 | Multiple types | 8 |
| **van der Kolk et al., 2007^124^**  **EMDR (8 sessions, 90 min.)**  **Placebo (8 sessions, 20-30 min.)** | 29  29 | 100 | CAPS | USA | 38.70 (14.30)  35.70 (13.40) | r.b.i. | Individual Individual | ITT | 76  86 | Multiple types | 8 |
| **van der Kolk et al., 2014^125^**  **YI** **(10 sessions, 60 min.)**  **HE (10 sessions, 60 min.)** | 32  32 | 100 | CAPS | USA | 41.50 (12.20)  44.30 (11.90) | n.a. | Group  Group | ITT | 100  100 | Multiple types | 5 |
| **van Gelderen et al., 2020^126^  3MDR (6 sessions, 70-90 min.)  NTCC** | 22  21 | 100 | CAPS | NL | 42.41 (9.80) 41.93 (9.12) | r.b.i. | Individual Individual | ITT | 4.5  0 | Multiple types | 7 |
| **Vaughan et al., 1994^127^  EMDR (3-5 sessions, 50 min.)  IHT (3-5 sessions, 50 min.)   AMR  WL** | 12  13  11  11 | 78 | SI-PTSD | Australia | 32.00 (14.70) | 3 | Individual  Individual  Individual | Compl. | 64 | Multiple types | 3 |
| **Wagner et al., 2019^128^  BA (8 sessions, 45 min.)  MDT (6 sessions, n.r.)** | 30 24 | 100 | CAPS | USA | 30.2 (6.4) 29.9 (7.1) | 3 | Individual  Combination | Compl. | 7 5 | Combat | 6 |
| **Wahbeh et al., 2016^129^  MM (6 sessions, 60 min.)  SQ (6 sessions, n.r.)** | 24  22 | 100 | PCL | Canada | 53.30 (12.60)  53.00 (11.80) | n.a. | Individual  Individual | Compl. | 7  4 | Combat | 4 |
| **Wells et al., 2012^130^  MCT (8 sessions, n.r.)**  **WL** | 10  10 | 100 | PDS | UK | 33.40 (13.40)  41.30 (13.70) | r.b.i. | Individual | ITT | 60  50 | Multiple types | 6 |
| **Wells et al., 2015^131^  MCT (8 sessions, 60 min.)  PE (8 sessions, 60 min.)**  **WL** | 10  10  10 | 100 | PDS | UK | 40.60 (11.90)  40.50 (10.90)  42.70 (18.50) | 3 | Individual  Individual | Compl. | 36  36  40 | Multiple types | 6 |
| **Yurtsever et al., 2018^132^**  **EMDR G-TEP (2 sessions, 240 min.) WL** | 18  29 | 100 | IES-R | Turkey (Syrian refugees) | 39.89 (10.96)  35.93 (11.10) | 1 | Group | Compl. | 72  79 | Multiple types | 5 |
| **Zang et al., 2013^133^  NET (4 sessions, 60-90 min.)  WL** | 11  11 | 100 | IES-R | China | 56.64 (12.22)  54.82 (11.59) | r.b.i. | Individual | ITT | 73  82 | Disaster | 7 |
| **Zang et al., 2014^134^  NET (4 sessions, 60-90 min.)  WL** | 10  10 | 100 | IES-R | China | 53.50 (1.24)  50.90 (1.23) | r.b.i. | Individual | ITT | 90  100 | Disaster | 7 |
| **Zlotnick et al., 1997^135^**  **AM (15 sessions, 120 min.) TAU (n.r.)** | 16  17 | 100 | DTS | USA | 39.00 (9.59) | n.a. | Group  Individual | Compl. | 100 | Childhood sexual abuse | 3 |
| **Zoellner et al., 2017^136^  IE + Placebo (5 sessions, 50 min.)  WL** | 16  11 | 100 | PSS-I | USA | 37.50 (12.40) | 3 | Individual | ITT | 71 | Multiple types | 6 |

Abbreviations*:* AL = Active Listening; AM = Affect-Management; AMR = Applied Muscle Relaxation; BA = Behavioral Activation; BEP = Brief Elective Psychotherapy; CA-CBT = Culturally Adapted Cognitive Behavioral Therapy; CAPS = Clinician-Administered PTSD Scale; CBCT = Cognitive-Behavior Couple Therapy; CBT = Cognitive-Behavioral Therapy; CM = Compassion Meditation; Compl. = Completer analysis; CPT = Cognitive-Processing Therapy; CR = Cognitive Restructuring; CT = Cognitive Therapy; CTT-BW = Cognitive Trauma-Therapy for Battered Women; DBT-PTSD = Dialectic Behavior Therapy for PTSD; DESTRESS-PC = Delivery of Self Training and Education for Stressful Situations-Primary Care version; DET = Dialogical Exposure Therapy; DRC = Democratic Republic of the Congo; DTS = Davidson Trauma Scale; EFST = Emotion Focused Supportive Therapy; EIT = Exposure Inhibition Therapy; EMDR = Eye Movement Desensitization and Reprocessing; EMDR G-TEP = EMDR Group Traumatic Episodic Protocol; EXP + CR = Exposure plus Cognitive Restructuring; EXP = Exposure; EXP+CR = Exposure plus Cognitive Restructuring; f2f = face to face; FSTTP = From Survivor to Thriver Program; GBET = Group-Based Exposure Therapy; GCBT = Group Cognitive Behavioral Therapy; GCPT = Group Cognitive-Processing Therapy; GPCT = Group Present-Centered Therapy; HE = Health Education; HOPE = Helping to Overcome PTSD through Empowerment; HTQ = Harvard Trauma Questionnaire; HYP = Holistic Yoga Program; I-CBT = Internet-based Cognitive Behavioral Therapy; IE = Imaginal Exposure; IE + Placebo = Imaginal Exposure plus pill placebo; IES = Impact of Event Scale; IES-R = Impact of Event Scale - Revised; IHT = Image Habituation Training; IN EXC = Integrative Exercise; IOK = Impact Of Killing; IPT = Inter-Personal Therapy; IPV = Intimate Partner Violence; IR = Imagery Rescripting; IRT = Imagery Rehearsal Therapy; I-SC = Internet-based Supportive Counseling; IT = Implosive Therapy; ITT = Intent-To-Treat analysis; MA = Minimal Attention; MCPT = Modified Cognitive-Processing Therapy; MCT = Meta-Cognitive Therapy; MDT = Multidisciplinary Treatment (specialist PTSD clinic); min. = minutes; MM = Mindfulness Meditation; MMPI = Minnesota Multiphasic Personality Inventory; MPSS = Modified PTSD Symptom Scale; M-PTSD = Mississippi scale for combat-related PTSD; MS = Multiple Sclerosis; n.a. = not applicable; NET = Narrative Exposure Therapy; NL = the Netherlands; n.r. = not reported; NTCC = Non-specific Treatment Component Control; PCL-5 = PTSD Checklist for DSM-5; PCL = PTSD Check-List – Civilian Version; PCL-M = PTSD Check-List - Military Version; PCT = Present-Centered Therapy; PDS = Posttraumatic Diagnostic Scale; PE = Prolonged Exposure; PE + SIT = Prolonged Exposure plus Stress Inoculation Training; PESHW = Psycho-educational self-help website; PFE = PTSD Family Education; Placebo = pill placebo control group; PMR = Progressive Muscle Relaxation; PSS-I = PTSD Symptom Scale – Interview; PSS-SR = PTSD Symptom Scale – Self-Report; PSY-EDU = Psychoeducation; PTSD-I = PTSD Interview; r.b.i. = reported but irrelevant (i.e., no meaningful group comparison possible at follow-up); ROT = Resilience Oriented Treatment; RT = Relaxation Therapy/Training; RTM = Reconsolidation of Traumatic Memories; SAT = Structured Approach Therapy; SC = Supportive Counselling; SE = Somatic Experiencing; SGT = Supportive Group Therapy; SI-PTSD = Structured Interview for PTSD; SIT = Stress Inoculation Training; SKY = Sudarshan Kriya Yoga; SMDT = Symptom-Monitoring Delayed Treatment group; SPT = Supportive Psychotherapy; SQ = Sitting Quietly; SSBT = Single-Session Behavioral Treatment; STAIR = Skills Training in Affective and Interpersonal Regulation; TARGET = Trauma Affect Regulation Guide for Education and Therapy; TAU = Treatment-As-Usual; TBG = Trauma and the Body Group; TC = Trauma Counselling; TF-CBT = Trauma-Focused Cognitive Behavioral Therapy; TFGT = Trauma-Focused Group Psychotherapy; TMed = Transcendental Meditation; TTP = Trauma Treatment Protocol; UK = United Kingdom; USA = United States of America; VC = Veteran.Calm; VRET = Virtual Reality Exposure Therapy; WL = Wait-List; WLP = Wellness Lifestyle Program; YI = Yoga Intervention.

^a^Subgroup sample size < 10 at post-treatment, hence, only FU results included in analyses and FU sample sizes reported.

^b^no post-treatment measure assessed, hence, FU sample sizes reported.
^c^FU results are based on PCL-C

| **Appendix B. Study quality** |  |  |  |  |  |  |  |  |  |
| --- | --- | --- | --- | --- | --- | --- | --- | --- | --- |
| **Trial** | **Q1 -PTSD** | **Q2 -manual** | **Q3 -training** | **Q4 -integrity** | **Q5 -ITT** | **Q6 –**  **N > 50** | **Q7 -random-ization** | **Q8 -blinding** | **Q sum score** |
| Acarturk et al. (2016) | 1 | 1 | 1 | 1 | 1 | 1 | 1 | 1 | 8 |
| Akbarian et al. (2015) | 1 | 1 | 1 | 0 | 1 | 0 | 0 | 1 | 5 |
| Asukai et al. (2010) | 1 | 1 | 1 | 1 | 1 | 0 | 1 | 1 | 7 |
| Basoglu et al. (2005) | 1 | 1 | 0 | 1 | 0 | 1 | 1 | 1 | 6 |
| Basoglu et al. (2007) | 1 | 1 | 1 | 0 | 1 | 0 | 1 | 1 | 6 |
| Beck et al. (2009) | 1 | 1 | 1 | 1 | 0 | 0 | 0 | 1 | 5 |
| Belleville et al. (2018) | 1 | 1 | 0 | 1 | 1 | 0 | 1 | 1 | 6 |
| Bisson et al. (2020) | 1 | 1 | 1 | 1 | 0 | 0 | 1 | 1 | 6 |
| Blanchard et al. (2003) | 0 | 1 | 1 | 1 | 1 | 1 | 0 | 1 | 6 |
| Bohus et al. (2013) | 1 | 1 | 1 | 1 | 1 | 1 | 0 | 1 | 7 |
| Bohus et al. (2020) | 1 | 1 | 1 | 1 | 1 | 1 | 1 | 1 | 8 |
| Bormann et al. (2008) | 1 | 1 | 0 | 1 | 0 | 0 | 1 | 1 | 5 |
| Bormann et al. (2013) | 1 | 0 | 0 | 1 | 1 | 1 | 1 | 1 | 6 |
| Boterhoven de Haan et al. (2020) | 1 | 1 | 1 | 1 | 0 | 1 | 1 | 1 | 7 |
| Brom et al. (2017) | 1 | 1 | 1 | 1 | 0 | 1 | 1 | 1 | 7 |
| Bryant et al. (2003) | 1 | 0 | 1 | 1 | 1 | 0 | 1 | 1 | 6 |
| Bryant et al. (2011) | 1 | 0 | 1 | 0 | 1 | 0 | 1 | 1 | 5 |
| Bryant et al. (2019) | 1 | 0 | 0 | 1 | 0 | 0 | 1 | 1 | 4 |
| Butollo et al. (2016) | 1 | 1 | 1 | 1 | 1 | 1 | 0 | 1 | 7 |
| Capezzani et al. (2013) | 1 | 0 | 1 | 0 | 1 | 0 | 0 | 1 | 4 |
| Carletto et al. (2016) | 1 | 1 | 1 | 1 | 0 | 0 | 1 | 1 | 6 |
| Carlson et al. (1998) | 1 | 1 | 1 | 1 | 0 | 0 | 0 | 0 | 4 |
| Carlsson et al. (2018) | 1 | 1 | 0 | 1 | 1 | 1 | 1 | 1 | 7 |
| Carter et al. (2013) | 1 | 1 | 1 | 0 | 0 | 0 | 1 | 1 | 5 |
| Castillo et al. (2016) | 1 | 1 | 0 | 1 | 1 | 1 | 0 | 1 | 6 |
| Chard (2005) | 1 | 1 | 1 | 1 | 0 | 1 | 0 | 1 | 6 |
| Classen et al. (2020) | 0 | 0 | 0 | 0 | 0 | 0 | 0 | 1 | 1 |
| Cloitre et al. (2002) | 1 | 1 | 1 | 1 | 0 | 0 | 0 | 1 | 5 |
| Cloitre et al. (2010) | 1 | 1 | 1 | 1 | 1 | 1 | 1 | 1 | 8 |
| Cottraux et al. (2008) | 1 | 1 | 1 | 1 | 0 | 0 | 1 | 1 | 6 |
| Davis et al. (2020) | 1 | 1 | 0 | 1 | 0 | 1 | 1 | 1 | 6 |
| Devilly et al. (1998) | 1 | 1 | 1 | 0 | 0 | 0 | 0 | 0 | 3 |
| Devilly et al. (1999) | 1 | 1 | 1 | 1 | 0 | 0 | 0 | 0 | 4 |
| Dorrepaal et al. (2012) | 1 | 1 | 0 | 0 | 1 | 1 | 1 | 1 | 6 |
| Duffy et al. (2007) | 1 | 1 | 1 | 0 | 1 | 1 | 1 | 1 | 7 |
| Dunne et al. (2012) | 1 | 1 | 1 | 0 | 1 | 0 | 0 | 0 | 4 |
| Echeburua et al. (1997) | 1 | 0 | 1 | 0 | 1 | 0 | 0 | 0 | 3 |
| Ehlers et al. (2003) | 1 | 1 | 0 | 1 | 1 | 1 | 1 | 1 | 7 |
| Ehlers et al. (2005) | 1 | 1 | 1 | 1 | 1 | 0 | 0 | 1 | 6 |
| Ehlers et al. (2014) | 1 | 1 | 1 | 1 | 1 | 1 | 1 | 1 | 8 |
| Engel et al. (2015) | 1 | 1 | 1 | 1 | 0 | 1 | 1 | 1 | 7 |
| Fecteau et al. (1999) | 1 | 1 | 1 | 1 | 0 | 0 | 1 | 1 | 6 |
| Foa et al. (1991) | 1 | 1 | 1 | 1 | 0 | 0 | 0 | 1 | 5 |
| Foa et al. (1999) | 1 | 1 | 1 | 1 | 0 | 0 | 0 | 1 | 5 |
| Foa et al. (2005) | 1 | 1 | 1 | 1 | 1 | 1 | 0 | 1 | 7 |
| Foa et al. (2018) | 1 | 1 | 1 | 1 | 1 | 1 | 1 | 1 | 8 |
| Forbes et al. (2012) | 1 | 1 | 0 | 1 | 1 | 1 | 1 | 1 | 7 |
| Ford et al. (2011) | 0 | 1 | 1 | 1 | 1 | 1 | 1 | 0 | 6 |
| Ford et al. (2013) | 0 | 1 | 1 | 1 | 0 | 1 | 1 | 1 | 6 |
| Galovski et al. (2012) | 1 | 1 | 1 | 1 | 1 | 1 | 1 | 1 | 8 |
| Gersons et al. (2000) | 1 | 1 | 1 | 1 | 1 | 0 | 1 | 1 | 7 |
| Ghafoori et al. (2017) | 1 | 0 | 1 | 1 | 1 | 1 | 1 | 0 | 6 |
| Goldstein et al. (2018) | 0 | 0 | 1 | 1 | 1 | 0 | 0 | 1 | 4 |
| Gray et al. (2019) | 1 | 1 | 0 | 1 | 1 | 1 | 1 | 1 | 7 |
| Hensel-Dittmann et al. (2011) | 1 | 1 | 1 | 1 | 0 | 0 | 1 | 1 | 6 |
| Hinton et al. (2005) | 1 | 1 | 1 | 1 | 1 | 0 | 1 | 1 | 7 |
| Hinton et al. (2011) | 1 | 1 | 1 | 0 | 1 | 0 | 0 | 1 | 5 |
| Hollifield et al. (2007) | 1 | 1 | 0 | 0 | 1 | 0 | 1 | 1 | 5 |
| Ivarsson et al. (2014) | 1 | 1 | 1 | 1 | 1 | 1 | 1 | 1 | 8 |
| Jacob et al. (2014) | 1 | 1 | 1 | 1 | 1 | 1 | 1 | 1 | 8 |
| Jalal et al. (2020) | 1 | 1 | 0 | 0 | 0 | 0 | 0 | 1 | 3 |
| Jensen et al. (1994) | 1 | 1 | 1 | 0 | 0 | 0 | 0 | 0 | 3 |
| Johnson et al. (2011) | 0 | 1 | 1 | 1 | 0 | 1 | 1 | 0 | 5 |
| Johnson et al. (2016) - post | 0 | 1 | 1 | 1 | 0 | 1 | 1 | 1 | 6 |
| Johnson et al. (2016) - FU2 | 0 | 1 | 1 | 1 | 0 | 0 | 1 | 1 | 5 |
| Keane et al. (1989) | 1 | 1 | 0 | 0 | 0 | 0 | 0 | 0 | 2 |
| Kent et al. (2011) | 1 | 0 | 1 | 0 | 1 | 0 | 0 | 1 | 4 |
| Krakow et al. (2000) | 0 | 1 | 0 | 0 | 0 | 1 | 0 | 1 | 3 |
| Krakow et al. (2001) | 0 | 1 | 0 | 0 | 0 | 1 | 1 | 1 | 4 |
| Krupnick et al. (2008) | 1 | 1 | 1 | 1 | 1 | 0 | 0 | 0 | 5 |
| Kubany et al. (2003) | 1 | 1 | 1 | 0 | 1 | 0 | 0 | 1 | 5 |
| Kubany et al. (2004) | 1 | 1 | 1 | 1 | 1 | 1 | 0 | 1 | 7 |
| Lang et al. (2019) | 1 | 1 | 1 | 0 | 0 | 0 | 1 | 1 | 5 |
| Langkaas et al. (2017) | 1 | 1 | 1 | 1 | 1 | 1 | 1 | 1 | 8 |
| Laugharne et al. (2016) | 1 | 1 | 1 | 1 | 1 | 0 | 1 | 1 | 7 |
| Lee et al. (2002) | 1 | 1 | 0 | 1 | 0 | 0 | 1 | 0 | 4 |
| Lely et al. (2019) | 1 | 1 | 1 | 1 | 1 | 0 | 1 | 1 | 7 |
| Lewis et al. (2017) | 1 | 1 | 1 | 1 | 1 | 0 | 1 | 1 | 7 |
| Lindauer et al. (2005) | 1 | 1 | 1 | 1 | 1 | 0 | 1 | 1 | 7 |
| Littleton et al. (2016) - post | 1 | 1 | 1 | 1 | 0 | 1 | 1 | 0 | 6 |
| Littleton et al. (2016) - FU1 | 1 | 1 | 1 | 1 | 0 | 0 | 1 | 0 | 5 |
| Litz et al. 2007 | 1 | 1 | 1 | 1 | 0 | 0 | 0 | 1 | 5 |
| Maguen et al. (2017) | 0 | 0 | 1 | 1 | 0 | 0 | 1 | 1 | 4 |
| Marcus et al. (1997) | 0 | 1 | 1 | 0 | 0 | 1 | 1 | 1 | 5 |
| Markowitz et al. (2015) | 1 | 0 | 1 | 1 | 1 | 1 | 1 | 1 | 7 |
| Marks et al. (1998) | 1 | 0 | 1 | 1 | 0 | 0 | 0 | 1 | 4 |
| McDonagh et al. (2005) | 1 | 1 | 1 | 1 | 1 | 0 | 0 | 1 | 6 |
| Mitchell et al. (2014) | 0 | 1 | 1 | 0 | 1 | 0 | 1 | 1 | 5 |
| Monson et al. (2006) | 1 | 1 | 1 | 1 | 1 | 1 | 0 | 1 | 7 |
| Monson et al. (2012) | 1 | 1 | 1 | 1 | 1 | 0 | 1 | 1 | 7 |
| Morath et al. (2014) | 1 | 1 | 1 | 1 | 0 | 0 | 1 | 1 | 6 |
| Mueser et al. (2008) | 1 | 0 | 0 | 1 | 0 | 1 | 1 | 1 | 5 |
| Nacasch et al. (2011) | 1 | 1 | 1 | 1 | 1 | 0 | 1 | 1 | 7 |
| Neuner et al. (2004) | 1 | 1 | 1 | 1 | 0 | 0 | 1 | 1 | 6 |
| Neuner et al. (2008) | 1 | 1 | 1 | 1 | 1 | 1 | 0 | 1 | 7 |
| Neuner et al. (2010) | 1 | 1 | 1 | 1 | 1 | 0 | 1 | 1 | 7 |
| Nidich et al. (2018) | 1 | 1 | 0 | 1 | 1 | 1 | 1 | 1 | 7 |
| Nijdam et al. (2012) | 1 | 1 | 1 | 1 | 0 | 1 | 1 | 1 | 7 |
| Niles et al. (2012) | 1 | 1 | 1 | 1 | 0 | 0 | 1 | 0 | 5 |
| Orang et al. (2018) | 1 | 1 | 1 | 0 | 0 | 0 | 1 | 1 | 5 |
| Pacella et al. (2012) | 1 | 1 | 1 | 1 | 1 | 1 | 1 | 1 | 8 |
| Paunovic (2011) | 1 | 0 | 1 | 0 | 0 | 0 | 0 | 0 | 2 |
| Power et al. (2002) - EMDR vs WL | 1 | 1 | 1 | 1 | 0 | 1 | 1 | 0 | 6 |
| Power et al. (2002) - all other comparisons | 1 | 1 | 1 | 1 | 0 | 0 | 1 | 0 | 5 |
| Rauch et al. (2015) | 1 | 1 | 1 | 0 | 0 | 0 | 0 | 1 | 4 |
| Ready et al. (2018) | 1 | 0 | 1 | 1 | 0 | 1 | 0 | 1 | 5 |
| Reger et al. (2016) | 1 | 1 | 1 | 1 | 1 | 1 | 1 | 1 | 8 |
| Resick et al. (2002) | 1 | 1 | 1 | 1 | 1 | 1 | 0 | 0 | 6 |
| Resick et al. (2015) | 1 | 0 | 1 | 1 | 1 | 1 | 0 | 1 | 6 |
| Robjant et al. (2019) | 1 | 1 | 1 | 1 | 0 | 1 | 1 | 1 | 7 |
| Rothbaum et al. (2005) | 1 | 1 | 1 | 1 | 0 | 0 | 0 | 1 | 5 |
| Sautter et al. (2015) | 1 | 1 | 1 | 1 | 1 | 1 | 0 | 1 | 7 |
| Schaal et al. (2009) | 1 | 1 | 1 | 1 | 1 | 0 | 1 | 1 | 7 |
| Scheck et al. (1998) | 0 | 1 | 1 | 0 | 0 | 1 | 1 | 1 | 5 |
| Schnurr et al. (2003) | 1 | 0 | 1 | 1 | 0 | 1 | 1 | 1 | 6 |
| Schnur et al. (2007) | 1 | 0 | 1 | 1 | 1 | 1 | 1 | 1 | 7 |
| Sloan et al. (2012) | 1 | 0 | 1 | 1 | 1 | 0 | 1 | 1 | 6 |
| Sloan et al. (2018) | 1 | 1 | 1 | 1 | 1 | 1 | 1 | 1 | 8 |
| Stanbury et al. (2020) | 1 | 1 | 1 | 1 | 0 | 0 | 0 | 1 | 5 |
| Stenmark et al. (2013) | 1 | 1 | 1 | 1 | 0 | 1 | 1 | 1 | 7 |
| Suris et al. (2013) | 1 | 1 | 1 | 1 | 0 | 1 | 1 | 1 | 7 |
| Taylor et al. (2003) | 1 | 1 | 1 | 1 | 1 | 0 | 0 | 1 | 6 |
| ter Heide et al. (2016) | 1 | 1 | 1 | 1 | 0 | 1 | 1 | 1 | 7 |
| Thorp et al. (2019) - post | 1 | 1 | 1 | 1 | 0 | 1 | 0 | 1 | 6 |
| Thorp et al. (2019) - FU2 | 1 | 1 | 1 | 1 | 0 | 0 | 0 | 1 | 5 |
| Tylee et al. (2017) | 1 | 1 | 0 | 1 | 1 | 0 | 0 | 1 | 5 |
| van den Burg et al. (2015) | 1 | 1 | 1 | 1 | 1 | 1 | 1 | 1 | 8 |
| van der Kolk et al. (2007) | 1 | 1 | 1 | 1 | 1 | 1 | 1 | 1 | 8 |
| van der Kolk et al. (2014) | 1 | 1 | 0 | 0 | 1 | 1 | 0 | 1 | 5 |
| van Gelderen et al. (2020) | 1 | 1 | 1 | 1 | 1 | 0 | 1 | 1 | 7 |
| Vaughan et al. (1994) | 0 | 1 | 1 | 0 | 0 | 0 | 0 | 1 | 3 |
| Wagner et al. (2019) | 1 | 1 | 1 | 1 | 0 | 1 | 0 | 1 | 6 |
| Wahbeh et al. (2016) | 1 | 1 | 0 | 0 | 0 | 0 | 1 | 0 | 3 |
| Wells et al. (2012) | 1 | 1 | 0 | 1 | 1 | 0 | 1 | 1 | 6 |
| Wells et al. (2015) | 1 | 1 | 1 | 1 | 0 | 0 | 1 | 1 | 6 |
| Yurtsever et al. (2018) | 1 | 1 | 1 | 0 | 0 | 0 | 1 | 1 | 5 |
| Zang et al. (2013) | 1 | 1 | 1 | 1 | 1 | 0 | 1 | 1 | 7 |
| Zang et al. (2014) | 1 | 1 | 1 | 1 | 1 | 0 | 1 | 1 | 7 |
| Zlotnick et al. (1997) | 1 | 0 | 1 | 0 | 0 | 0 | 0 | 1 | 3 |
| Zoellner et al. (2017) | 1 | 1 | 1 | 1 | 1 | 0 | 1 | 0 | 6 |

**Appendix C. References of the included studies**

References

1. Acarturk C, Konuk E, Cetinkaya M, et al. The efficacy of eye movement desensitization and reprocessing for post-traumatic stress disorder and depression among Syrian refugees: results of a randomized controlled trial. *Psychol Med*. 2016;46(12):2583-2593. doi:10.1017/S0033291716001070.

2. Akbarian F, Bajoghli H, Haghighi M, Kalak N, Holsboer-Trachsler E, Brand S. The effectiveness of cognitive behavioral therapy with respect to psychological symptoms and recovering autobiographical memory in patients suffering from post-traumatic stress disorder. *Neuropsychiatr Dis Treat*. 2015;11(19):395-404. doi:10.2147/NDT.S79581.

3. Asukai N, Saito A, Tsuruta N, Kishimoto J, Nishikawa T. Efficacy of exposure therapy for Japanese patients with posttraumatic stress disorder due to mixed traumatic events: A randomized controlled study. *J Trauma Stress*. 2010;23(6):744-750. doi:10.1002/jts.20589.

4. Başoğlu M, Salcioğlu E, Livanou M, Kalender D, Acar G. Single-session behavioral treatment of earthquake-related posttraumatic stress disorder: a randomized waiting list controlled trial. *J Trauma Stress*. 2005;18(1):1-11. doi:10.1002/jts.20011.

5. Başoglu M, Salcioglu E, Livanou M. A randomized controlled study of single-session behavioural treatment of earthquake-related post-traumatic stress disorder using an earthquake simulator. *Psychol Med*. 2007;37(2):203-213. doi:10.1017/S0033291706009123.

6. Beck JG, Coffey SF, Foy DW, Keane TM, Blanchard EB. Group cognitive behavior therapy for chronic posttraumatic stress disorder: an initial randomized pilot study. *Behav Ther*. 2009;40(1):82-92. doi:10.1016/j.beth.2008.01.003.

7. Belleville G, Dubé-Frenette M, Rousseau A. Efficacy of Imagery Rehearsal Therapy and Cognitive Behavioral Therapy in Sexual Assault Victims With Posttraumatic Stress Disorder: A Randomized Controlled Trial. *J Trauma Stress*. 2018;31(4):591-601. doi:10.1002/jts.22306.

8. Bisson JI, van Deursen R, Hannigan B, et al. Randomized controlled trial of multi-modular motion-assisted memory desensitization and reconsolidation (3MDR) for male military veterans with treatment-resistant post-traumatic stress disorder. *Acta Psychiatr Scand*. 2020;142(2):141-151. doi:10.1111/acps.13200.

9. Blanchard EB, Hickling EJ, Devineni T, et al. A controlled evaluation of cognitive behaviorial therapy for posttraumatic stress in motor vehicle accident survivors. *Behav Res Ther*. 2003;41(1):79-96. doi:10.1016/S0005-7967(01)00131-0.

10. Bohus M, Dyer AS, Priebe K, et al. Dialectical behaviour therapy for post-traumatic stress disorder after childhood sexual abuse in patients with and without borderline personality disorder: a randomised controlled trial. *Psychother Psychosom*. 2013;82(4):221-233. doi:10.1159/000348451.

11. Bohus M, Kleindienst N, Hahn C, et al. Dialectical Behavior Therapy for Posttraumatic Stress Disorder (DBT-PTSD) Compared With Cognitive Processing Therapy (CPT) in Complex Presentations of PTSD in Women Survivors of Childhood Abuse: A Randomized Clinical Trial. *JAMA Psychiatry*. 2020;(77):1235-1245. doi:10.1001/jamapsychiatry.2020.2148.

12. Bormann JE, Thorp S, Wetherell JL, Golshan S. A spiritually based group intervention for combat veterans with posttraumatic stress disorder: feasibility study. *J Holist Nurs*. 2008;26(2):109-116. doi:10.1177/0898010107311276.

13. Bormann JE, Thorp SR, Wetherell JL, Golshan S, Lang AJ. Meditation-based mantram intervention for veterans with posttraumatic stress disorder: A randomized trial. *Psychol Trauma*. 2013;5(3):259-267. doi:10.1037/a0027522.

14. Boterhoven de Haan KL, Lee CW, Fassbinder E, et al. Imagery rescripting and eye movement desensitisation and reprocessing as treatment for adults with post-traumatic stress disorder from childhood trauma: randomised clinical trial. *Br J Psychiatry*. 2020;217(5):609-615. doi:10.1192/bjp.2020.158.

15. Brom D, Stokar Y, Lawi C, et al. Somatic Experiencing for Posttraumatic Stress Disorder: A Randomized Controlled Outcome Study. *J Trauma Stress*. 2017;30(3):304-312. doi:10.1002/jts.22189.

16. Bryant RA, Moulds ML, Guthrie RM, Dang ST, Nixon RDV. Imaginal exposure alone and imaginal exposure with cognitive restructuring in treatment of posttraumatic stress disorder. *J Consult Clin Psychol*. 2003;71(4):706-712. doi:10.1037/0022-006x.71.4.706.

17. Bryant RA, Ekasawin S, Chakrabhand S, Suwanmitri S, Duangchun O, Chantaluckwong T. A randomized controlled effectiveness trial of cognitive behavior therapy for post-traumatic stress disorder in terrorist-affected people in Thailand. *World Psychiatry*. 2011;10(3):205-209. doi:10.1002/j.2051-5545.2011.tb00058.x.

18. Bryant RA, Kenny L, Rawson N, et al. Efficacy of exposure-based cognitive behaviour therapy for post-traumatic stress disorder in emergency service personnel: a randomised clinical trial. *Psychol Med*. 2019;49(9):1565-1573. doi:10.1017/S0033291718002234.

19. Butollo W, Karl R, König J, Rosner R. A Randomized Controlled Clinical Trial of Dialogical Exposure Therapy versus Cognitive Processing Therapy for Adult Outpatients Suffering from PTSD after Type I Trauma in Adulthood. *Psychother Psychosom*. 2016;85(1):16-26. doi:10.1159/000440726.

20. Capezzani L, Ostacoli L, Cavallo M, et al. EMDR and CBT for Cancer Patients: Comparative Study of Effects on PTSD, Anxiety, and Depression. *J EMDR Prac Res*. 2013;7(3):134-143. doi:10.1891/1933-3196.7.3.134.

21. Carletto S, Borghi M, Bertino G, et al. Treating Post-traumatic Stress Disorder in Patients with Multiple Sclerosis: A Randomized Controlled Trial Comparing the Efficacy of Eye Movement Desensitization and Reprocessing and Relaxation Therapy. *Front Psychol*. 2016;7(21):526. doi:10.3389/fpsyg.2016.00526.

22. Carlson JG, Chemtob CM, Rusnak K, Hedlund NL, Muraoka MY. Eye movement desensitization and reprocessing (EDMR) treatment for combat-related posttraumatic stress disorder. *J Trauma Stress*. 1998;11(1):3-24. doi:10.1023/A:1024448814268.

23. Carlsson J, Sonne C, Vindbjerg E, Mortensen EL. Stress management versus cognitive restructuring in trauma-affected refugees-A pragmatic randomised study. *Psychiatry Res*. 2018;266:116-123. doi:10.1016/j.psychres.2018.05.015.

24. J.Carter J, L.Gerbarg P, Brown R, Ware R, Katzman M. Multi-Component Yoga Breath Program for Vietnam Veteran Post Traumatic Stress Disorder: Randomized Controlled Trial. *J Trauma Stress Disor Treat*. 2013;02(03). doi:10.4172/2324-8947.1000108.

25. Castillo DT, Chee CL, Nason E, et al. Group-delivered cognitive/exposure therapy for PTSD in women veterans: A randomized controlled trial. *Psychol Trauma*. 2016;8(3):404-412. doi:10.1037/tra0000111.

26. Chard KM. An evaluation of cognitive processing therapy for the treatment of posttraumatic stress disorder related to childhood sexual abuse. *J Consult Clin Psychol*. 2005;73(5):965-971. doi:10.1037/0022-006X.73.5.965.

27. Classen CC, Hughes L, Clark C, Hill Mohammed B, Woods P, Beckett B. A Pilot RCT of A Body-Oriented Group Therapy For Complex Trauma Survivors: An Adaptation of Sensorimotor Psychotherapy. *J Trauma Dissociation*. 2021;22(1):52-68. doi:10.1080/15299732.2020.1760173.

28. Cloitre M, Koenen KC, Cohen LR, Han H. Skills training in affective and interpersonal regulation followed by exposure: A phase-based treatment for PTSD related to childhood abuse. *J Consult Clin Psychol*. 2002;70(5):1067-1074. doi:10.1037//0022-006X.70.5.1067.

29. Cloitre M, Stovall-McClough KC, Nooner K, et al. Treatment for PTSD related to childhood abuse: a randomized controlled trial. *Am J Psychiatry*. 2010;167(8):915-924. doi:10.1176/appi.ajp.2010.09081247.

30. Cottraux J, Note I, Yao SN, et al. Randomized controlled comparison of cognitive behavior therapy with Rogerian supportive therapy in chronic post-traumatic stress disorder: a 2-year follow-up. *Psychother Psychosom*. 2008;77(2):101-110. doi:10.1159/000112887.

31. Davis LW, Schmid AA, Daggy JK, et al. Symptoms improve after a yoga program designed for PTSD in a randomized controlled trial with veterans and civilians. *Psychol Trauma*. 2020;12(8):904-912. doi:10.1037/tra0000564.

32. Devilly GJ, Spence SH, Rapee RM. Statistical and reliable change with eye movement desensitization and reprocessing: Treating trauma within a veteran population. *Behav Ther*. 1998;29(3):435-455. doi:10.1016/S0005-7894(98)80042-7.

33. Devilly GJ, Spence SH. The Relative Efficacy and Treatment Distress of EMDR and a Cognitive-Behavior Trauma Treatment Protocol in the Amelioration of Posttraumatic Stress Disorder. *J Anxiety Disord*. 1999;13(1-2):131-157. doi:10.1016/s0887-6185(98)00044-9.

34. Dorrepaal E, Thomaes K, Smit JH, et al. Stabilizing group treatment for complex posttraumatic stress disorder related to child abuse based on psychoeducation and cognitive behavioural therapy: a multisite randomized controlled trial. *Psychother Psychosom*. 2012;81(4):217-225. doi:10.1159/000335044.

35. Duffy M, Gillespie K, Clark DM. Post-traumatic stress disorder in the context of terrorism and other civil conflict in Northern Ireland: randomised controlled trial. *BMJ*. 2007;334(7604):1147. doi:10.1136/bmj.39021.846852.BE.

36. Dunne RL, Kenardy J, Sterling M. A randomized controlled trial of cognitive-behavioral therapy for the treatment of PTSD in the context of chronic whiplash. *Clin J Pain*. 2012;28(9):755-765. doi:10.1097/AJP.0b013e318243e16b.

37. Echeburúa E, Corral P de, Zubizarreta I, Sarasua B. Psychological treatment of chronic posttraumatic stress disorder in victims of sexual aggression. *Behav Modif*. 1997;21(4):433-456. doi:10.1177/01454455970214003.

38. Ehlers A, Clark DM, Hackmann A, et al. A randomized controlled trial of cognitive therapy, a self-help booklet, and repeated assessments as early interventions for posttraumatic stress disorder. *Arch Gen Psychiatry*. 2003;60(10):1024-1032. doi:10.1001/archpsyc.60.10.1024.

39. Ehlers A, Clark DM, Hackmann A, McManus F, Fennell M. Cognitive therapy for post-traumatic stress disorder: development and evaluation. *Behav Res Ther*. 2005;43(4):413-431. doi:10.1016/j.brat.2004.03.006.

40. Ehlers A, Hackmann A, Grey N, et al. A randomized controlled trial of 7-day intensive and standard weekly cognitive therapy for PTSD and emotion-focused supportive therapy. *Am J Psychiatry*. 2014;171(3):294-304. doi:10.1176/appi.ajp.2013.13040552.

41. Engel CC, Litz B, Magruder KM, et al. Delivery of self training and education for stressful situations (DESTRESS-PC): a randomized trial of nurse assisted online self-management for PTSD in primary care. *Gen Hosp Psychiatry*. 2015;37(4):323-328. doi:10.1016/j.genhosppsych.2015.04.007.

42. Fecteau G, Nicki R. COGNITIVE BEHAVIOURAL TREATMENT OF POST TRAUMATIC STRESS DISORDER AFTER MOTOR VEHICLE ACCIDENT. *Behav Cogn Psychother*. 1999;27(3):201-214. doi:10.1017/S135246589927302X.

43. Foa EB, Rothbaum BO, Riggs DS, Murdock TB. Treatment of posttraumatic stress disorder in rape victims: A comparison between cognitive-behavioral procedures and counseling. *J Consult Clin Psychol*. 1991;59(5):715-723. doi:10.1037//0022-006x.59.5.715.

44. Foa EB, Dancu CV, Hembree EA, Jaycox LH, Meadows EA, Street GP. A comparison of exposure therapy, stress inoculation training, and their combination for reducing posttraumatic stress disorder in female assault victims. *J Consult Clin Psychol*. 1999;67(2):194-200. doi:10.1037//0022-006x.67.2.194.

45. Foa EB, Hembree EA, Cahill SP, et al. Randomized trial of prolonged exposure for posttraumatic stress disorder with and without cognitive restructuring: outcome at academic and community clinics. *J Consult Clin Psychol*. 2005;73(5):953-964. doi:10.1037/0022-006X.73.5.953.

46. Foa EB, McLean CP, Zang Y, et al. Effect of Prolonged Exposure Therapy Delivered Over 2 Weeks vs 8 Weeks vs Present-Centered Therapy on PTSD Symptom Severity in Military Personnel: A Randomized Clinical Trial. *JAMA*. 2018;319(4):354-364. doi:10.1001/jama.2017.21242.

47. Forbes D, Lloyd D, Nixon RDV, et al. A multisite randomized controlled effectiveness trial of cognitive processing therapy for military-related posttraumatic stress disorder. *J Anxiety Disord*. 2012;26(3):442-452. doi:10.1016/j.janxdis.2012.01.006.

48. Ford JD, Steinberg KL, Zhang W. A randomized clinical trial comparing affect regulation and social problem-solving psychotherapies for mothers with victimization-related PTSD. *Behav Ther*. 2011;42(4):560-578. doi:10.1016/j.beth.2010.12.005.

49. Ford JD, Chang R, Levine J, Zhang W. Randomized clinical trial comparing affect regulation and supportive group therapies for victimization-related PTSD with incarcerated women. *Behav Ther*. 2013;44(2):262-276. doi:10.1016/j.beth.2012.10.003.

50. Galovski TE, Blain LM, Mott JM, Elwood L, Houle T. Manualized therapy for PTSD: flexing the structure of cognitive processing therapy. *J Consult Clin Psychol*. 2012;80(6):968-981. doi:10.1037/a0030600.

51. Gersons BP, Carlier IV, Lamberts RD, van der Kolk, B A. Randomized clinical trial of brief eclectic psychotherapy for police officers with posttraumatic stress disorder. *J Trauma Stress*. 2000;13(2):333-347. doi:10.1023/A:1007793803627.

52. Ghafoori B, Hansen MC, Garibay E, Korosteleva O. Feasibility of Training Frontline Therapists in Prolonged Exposure: A Randomized Controlled Pilot Study of Treatment of Complex Trauma in Diverse Victims of Crime and Violence. *J Nerv Ment Dis*. 2017;205(4):283-293. doi:10.1097/NMD.0000000000000659.

53. Goldstein LA, Mehling WE, Metzler TJ, et al. Veterans Group Exercise: A randomized pilot trial of an Integrative Exercise program for veterans with posttraumatic stress. *J Affect Disord*. 2018;227:345-352. doi:10.1016/j.jad.2017.11.002.

54. Gray R, Budden-Potts D, Bourke F. Reconsolidation of Traumatic Memories for PTSD: A randomized controlled trial of 74 male veterans. *Psychother Res*. 2019;29(5):621-639. doi:10.1080/10503307.2017.1408973.

55. Hensel-Dittmann D, Schauer M, Ruf M, et al. Treatment of traumatized victims of war and torture: a randomized controlled comparison of narrative exposure therapy and stress inoculation training. *Psychother Psychosom*. 2011;80(6):345-352. doi:10.1159/000327253.

56. Hinton DE, Chhean D, Pich V, Safren SA, Hofmann SG, Pollack MH. A randomized controlled trial of cognitive-behavior therapy for Cambodian refugees with treatment-resistant PTSD and panic attacks: a cross-over design. *J Trauma Stress*. 2005;18(6):617-629. doi:10.1002/jts.20070.

57. Hinton DE, Hofmann SG, Rivera E, Otto MW, Pollack MH. Culturally adapted CBT (CA-CBT) for Latino women with treatment-resistant PTSD: a pilot study comparing CA-CBT to applied muscle relaxation. *Behav Res Ther*. 2011;49(4):275-280. doi:10.1016/j.brat.2011.01.005.

58. Hollifield M, Sinclair-Lian N, Warner TD, Hammerschlag R. Acupuncture for posttraumatic stress disorder: a randomized controlled pilot trial. *J Nerv Ment Dis*. 2007;195(6):504-513. doi:10.1097/NMD.0b013e31803044f8.

59. Ivarsson D, Blom M, Hesser H, et al. Guided internet-delivered cognitive behavior therapy for post-traumatic stress disorder: A randomized controlled trial. *Internet Interventions*. 2014;1(1):33-40. doi:10.1016/j.invent.2014.03.002.

60. Jacob N, Neuner F, Maedl A, Schaal S, Elbert T. Dissemination of psychotherapy for trauma spectrum disorders in postconflict settings: a randomized controlled trial in Rwanda. *Psychother Psychosom*. 2014;83(6):354-363. doi:10.1159/000365114.

61. Jalal B, Kruger Q, Hinton DE, others. Culturally adapted CBT (CA-CBT) for traumatised indigenous South Africans (Sepedi): a randomised pilot trial comparing CA-CBT to applied muscle relaxation. *Intervention*. 2020;18:61-65.

62. Jensen JA. An investigation of eye movement desensitization and reprocessing (EMD/R) as a treatment for posttraumatic stress disorder (PTSD) symptoms of Vietnam combat veterans. *Behav Ther*. 1994;25(2):311-325. doi:10.1016/S0005-7894(05)80290-4.

63. Johnson DM, Zlotnick C, Perez S. Cognitive behavioral treatment of PTSD in residents of battered women's shelters: results of a randomized clinical trial. *J Consult Clin Psychol*. 2011;79(4):542-551. doi:10.1037/a0023822.

64. Johnson DM, Johnson NL, Perez SK, Palmieri PA, Zlotnick C. Comparison of Adding Treatment of PTSD During and After Shelter Stay to Standard Care in Residents of Battered Women's Shelters: Results of a Randomized Clinical Trial. *J Trauma Stress*. 2016;29(4):365-373. doi:10.1002/jts.22117.

65. Keane TM, Fairbank JA, Caddell JM, Zimering RT. Implosive (flooding) therapy reduces symptoms of PTSD in Vietnam combat veterans. *Behav Ther*. 1989;20(2):245-260. doi:10.1016/S0005-7894(89)80072-3.

66. Kent M, Davis MC, Stark SL, Stewart LA. A resilience-oriented treatment for posttraumatic stress disorder: results of a preliminary randomized clinical trial. *J Trauma Stress*. 2011;24(5):591-595. doi:10.1002/jts.20685.

67. Krakow B, Hollifield M, Schrader R, et al. A controlled study of imagery rehearsal for chronic nightmares in sexual assault survivors with PTSD: a preliminary report. *J Trauma Stress*. 2000;13(4):589-609. doi:10.1023/A:1007854015481.

68. Krakow B, Hollifield M, Johnston L, et al. Imagery rehearsal therapy for chronic nightmares in sexual assault survivors with posttraumatic stress disorder: a randomized controlled trial. *JAMA*. 2001;286(5):537-545. doi:10.1001/jama.286.5.537.

69. Krupnick JL, Green BL, Stockton P, Miranda J, Krause E, Mete M. Group interpersonal psychotherapy for low-income women with posttraumatic stress disorder. *Psychother Res*. 2008;18(5):497-507. doi:10.1080/10503300802183678.

70. Kubany ES, Hill EE, Owens JA. Cognitive trauma therapy for battered women with PTSD: preliminary findings. *J Trauma Stress*. 2003;16(1):81-91. doi:10.1023/A:1022019629803.

71. Kubany ES, Hill EE, Owens JA, et al. Cognitive trauma therapy for battered women with PTSD (CTT-BW). *J Consult Clin Psychol*. 2004;72(1):3-18. doi:10.1037/0022-006X.72.1.3.

72. Lang AJ, Malaktaris AL, Casmar P, et al. Compassion Meditation for Posttraumatic Stress Disorder in Veterans: A Randomized Proof of Concept Study. *J Trauma Stress*. 2019;32(2):299-309. doi:10.1002/jts.22397.

73. Langkaas TF, Hoffart A, Øktedalen T, Ulvenes PG, Hembree EA, Smucker M. Exposure and non-fear emotions: A randomized controlled study of exposure-based and rescripting-based imagery in PTSD treatment. *Behav Res Ther*. 2017;97:33-42. doi:10.1016/j.brat.2017.06.007.

74. Laugharne J, Kullack C, Lee CW, et al. Amygdala Volumetric Change Following Psychotherapy for Posttraumatic Stress Disorder. *J Neuropsychiatry Clin Neurosci*. 2016;28(4):312-318. doi:10.1176/appi.neuropsych.16010006.

75. Lee C, Gavriel H, Drummond P, Richards J, Greenwald R. Treatment of PTSD: stress inoculation training with prolonged exposure compared to EMDR. *J Clin Psychol*. 2002;58(9):1071-1089. doi:10.1002/jclp.10039.

76. Lely JCG, Knipscheer JW, Moerbeek M, Ter Heide, F J J, van den Bout J, Kleber RJ. Randomised controlled trial comparing narrative exposure therapy with present-centred therapy for older patients with post-traumatic stress disorder. *Br J Psychiatry*. 2019;214(6):369-377. doi:10.1192/bjp.2019.59.

77. Lewis CE, Farewell D, Groves V, et al. Internet-based guided self-help for posttraumatic stress disorder (PTSD): Randomized controlled trial. *Depress Anxiety*. 2017;34(6):555-565. doi:10.1002/da.22645.

78. Lindauer RJL, Gersons BPR, van Meijel, Els P M, et al. Effects of brief eclectic psychotherapy in patients with posttraumatic stress disorder: randomized clinical trial. *J Trauma Stress*. 2005;18(3):205-212. doi:10.1002/jts.20029.

79. Littleton H, Grills AE, Kline KD, Schoemann AM, Dodd JC. The From Survivor to Thriver program: RCT of an online therapist-facilitated program for rape-related PTSD. *J Anxiety Disord*. 2016;43:41-51.

80. Litz BT, Engel CC, Bryant RA, Papa A. A randomized, controlled proof-of-concept trial of an Internet-based, therapist-assisted self-management treatment for posttraumatic stress disorder. *Am J Psychiatry*. 2007;164(11):1676-1683. doi:10.1176/appi.ajp.2007.06122057.

81. Maguen S, Burkman K, Madden E, et al. Impact of Killing in War: A Randomized, Controlled Pilot Trial. *J Clin Psychol*. 2017;73(9):997-1012. doi:10.1002/jclp.22471.

82. Marcus SV, Marquis P, Sakai C. Controlled study of treatment of PTSD using EMDR in an HMO setting. *Psychotherapy: Theory, Research, Practice, Training*. 1997;34(3):307-315. doi:10.1037/h0087791.

83. Markowitz JC, Petkova E, Neria Y, et al. Is Exposure Necessary? A Randomized Clinical Trial of Interpersonal Psychotherapy for PTSD. *Am J Psychiatry*. 2015;172(5):430-440. doi:10.1176/appi.ajp.2014.14070908.

84. Marks I, Lovell K, Noshirvani H, Livanou M, Thrasher S. Treatment of posttraumatic stress disorder by exposure and/or cognitive restructuring: a controlled study. *Arch Gen Psychiatry*. 1998;55(4):317-325. doi:10.1001/archpsyc.55.4.317.

85. McDonagh A, Friedman M, McHugo G, et al. Randomized trial of cognitive-behavioral therapy for chronic posttraumatic stress disorder in adult female survivors of childhood sexual abuse. *J Consult Clin Psychol*. 2005;73(3):515-524. doi:10.1037/0022-006X.73.3.515.

86. Mitchell KS, Dick AM, DiMartino DM, et al. A pilot study of a randomized controlled trial of yoga as an intervention for PTSD symptoms in women. *J Trauma Stress*. 2014;27(2):121-128. doi:10.1002/jts.21903.

87. Monson CM, Schnurr PP, Resick PA, Friedman MJ, Young-Xu Y, Stevens SP. Cognitive processing therapy for veterans with military-related posttraumatic stress disorder. *J Consult Clin Psychol*. 2006;74(5):898-907. doi:10.1037/0022-006X.74.5.898.

88. Monson CM, Fredman SJ, Macdonald A, Pukay-Martin ND, Resick PA, Schnurr PP. Effect of cognitive-behavioral couple therapy for PTSD: a randomized controlled trial. *JAMA*. 2012;308(7):700-709. doi:10.1001/jama.2012.9307.

89. Morath J, Moreno-Villanueva M, Hamuni G, et al. Effects of psychotherapy on DNA strand break accumulation originating from traumatic stress. *Psychother Psychosom*. 2014;83(5):289-297. doi:10.1159/000362739.

90. Mueser KT, Rosenberg SD, Xie H, et al. A randomized controlled trial of cognitive-behavioral treatment for posttraumatic stress disorder in severe mental illness. *J Consult Clin Psychol*. 2008;76(2):259-271. doi:10.1037/0022-006X.76.2.259.

91. Nacasch N, Foa EB, Huppert JD, et al. Prolonged exposure therapy for combat- and terror-related posttraumatic stress disorder: a randomized control comparison with treatment as usual. *J Clin Psychiatry*. 2011;72(9):1174-1180. doi:10.4088/JCP.09m05682blu.

92. Neuner F, Schauer M, Klaschik C, Karunakara U, Elbert T. A comparison of narrative exposure therapy, supportive counseling, and psychoeducation for treating posttraumatic stress disorder in an african refugee settlement. *J Consult Clin Psychol*. 2004;72(4):579-587. doi:10.1037/0022-006X.72.4.579.

93. Neuner F, Onyut PL, Ertl V, Odenwald M, Schauer E, Elbert T. Treatment of posttraumatic stress disorder by trained lay counselors in an African refugee settlement: a randomized controlled trial. *J Consult Clin Psychol*. 2008;76(4):686-694. doi:10.1037/0022-006X.76.4.686.

94. Neuner F, Kurreck S, Ruf M, Odenwald M, Elbert T, Schauer M. Can asylum-seekers with posttraumatic stress disorder be successfully treated? A randomized controlled pilot study. *Cogn Behav Ther*. 2010;39(2):81-91. doi:10.1080/16506070903121042.

95. Nidich S, Mills PJ, Rainforth M, et al. Non-trauma-focused meditation versus exposure therapy in veterans with post-traumatic stress disorder: A randomised controlled trial. *Lancet Psychiatry*. 2018;5(12):975-986. doi:10.1016/S2215-0366(18)30384-5.

96. Nijdam MJ, Gersons BPR, Reitsma JB, Jongh A de, Olff M. Brief eclectic psychotherapy v. eye movement desensitisation and reprocessing therapy for post-traumatic stress disorder: randomised controlled trial. *Br J Psychiatry*. 2012;200(3):224-231. doi:10.1192/bjp.bp.111.099234.

97. Niles BL, Klunk-Gillis J, Ryngala DJ, Silberbogen AK, Paysnick A, Wolf EJ. Comparing mindfulness and psychoeducation treatments for combat-related PTSD using a telehealth approach. *Psychol Trauma*. 2012;4(5):538-547. doi:10.1037/a0026161.

98. Orang T, Ayoughi S, Moran JK, et al. The efficacy of narrative exposure therapy in a sample of Iranian women exposed to ongoing intimate partner violence-A randomized controlled trial. *Clin Psychol Psychother*. 2018;25(6):827-841. doi:10.1002/cpp.2318.

99. Pacella ML, Armelie A, Boarts J, et al. The impact of prolonged exposure on PTSD symptoms and associated psychopathology in people living with HIV: a randomized test of concept. *AIDS Behav*. 2012;16(5):1327-1340. doi:10.1007/s10461-011-0076-y.

100. Paunović N. Exposure Inhibition Therapy as a Treatment for Chronic Posttraumatic Stress Disorder: A Controlled Pilot Study. *PSYCH*. 2011;02(06):605-614. doi:10.4236/psych.2011.26093.

101. Power K, McGoldrick T, Brown K, et al. A controlled comparison of eye movement desensitization and reprocessing versus exposure plus cognitive restructuring versus waiting list in the treatment of post-traumatic stress disorder. *Clin Psychol Psychother*. 2002;9(5):299-318. doi:10.1002/cpp.341.

102. Rauch SAM, King AP, Abelson J, et al. Biological and symptom changes in posttraumatic stress disorder treatment: a randomized clinical trial. *Depress Anxiety*. 2015;32(3):204-212. doi:10.1002/da.22331.

103. Ready DJ, Mascaro N, Wattenberg MS, Sylvers P, Worley V, Bradley-Davino B. A Controlled Study of Group-Based Exposure Therapy with Vietnam-Era Veterans. *Journal of Loss and Trauma*. 2018;23(6):439-457. doi:10.1080/15325024.2018.1485268.

104. Reger GM, Koenen-Woods P, Zetocha K, et al. Randomized controlled trial of prolonged exposure using imaginal exposure vs. virtual reality exposure in active duty soldiers with deployment-related posttraumatic stress disorder (PTSD). *J Consult Clin Psychol*. 2016;84(11):946-959. doi:10.1037/ccp0000134.

105. Resick PA, Nishith P, Weaver TL, Astin MC, Feuer CA. A comparison of cognitive-processing therapy with prolonged exposure and a waiting condition for the treatment of chronic posttraumatic stress disorder in female rape victims. *J Consult Clin Psychol*. 2002;70(4):867-879. doi:10.1037//0022-006X.70.4.867.

106. Resick PA, Wachen JS, Mintz J, et al. A randomized clinical trial of group cognitive processing therapy compared with group present-centered therapy for PTSD among active duty military personnel. *J Consult Clin Psychol*. 2015;83(6):1058-1068. doi:10.1037/ccp0000016.

107. Robjant K, Koebach A, Schmitt S, Chibashimba A, Carleial S, Elbert T. The treatment of posttraumatic stress symptoms and aggression in female former child soldiers using adapted Narrative Exposure therapy - a RCT in Eastern Democratic Republic of Congo. *Behav Res Ther*. 2019;123:103482. doi:10.1016/j.brat.2019.103482.

108. Rothbaum BO, Astin MC, Marsteller F. Prolonged Exposure versus Eye Movement Desensitization and Reprocessing (EMDR) for PTSD rape victims. *J Trauma Stress*. 2005;18(6):607-616. doi:10.1002/jts.20069.

109. Sautter FJ, Glynn SM, Cretu JB, Senturk D, Vaught AS. Efficacy of structured approach therapy in reducing PTSD in returning veterans: A randomized clinical trial. *Psychol Serv*. 2015;12(3):199-212. doi:10.1037/ser0000032.

110. Schaal S, Elbert T, Neuner F. Narrative exposure therapy versus interpersonal psychotherapy. A pilot randomized controlled trial with Rwandan genocide orphans. *Psychother Psychosom*. 2009;78(5):298-306. doi:10.1159/000229768.

111. Scheck MM, Schaeffer JA, Gillette C. Brief psychological intervention with traumatized young women: the efficacy of eye movement desensitization and reprocessing. *J Trauma Stress*. 1998;11(1):25-44. doi:10.1023/A:1024400931106.

112. Schnurr PP, Friedman MJ, Foy DW, et al. Randomized trial of trauma-focused group therapy for posttraumatic stress disorder: results from a department of veterans affairs cooperative study. *Arch Gen Psychiatry*. 2003;60(5):481-489. doi:10.1001/archpsyc.60.5.481.

113. Schnurr PP, Friedman MJ, Engel CC, et al. Cognitive behavioral therapy for posttraumatic stress disorder in women: a randomized controlled trial. *JAMA*. 2007;297(8):820-830. doi:10.1001/jama.297.8.820.

114. Sloan DM, Marx BP, Bovin MJ, Feinstein BA, Gallagher MW. Written exposure as an intervention for PTSD: a randomized clinical trial with motor vehicle accident survivors. *Behav Res Ther*. 2012;50(10):627-635. doi:10.1016/j.brat.2012.07.001.

115. Sloan DM, Unger W, Lee DJ, Beck JG. A Randomized Controlled Trial of Group Cognitive Behavioral Treatment for Veterans Diagnosed With Chronic Posttraumatic Stress Disorder. *J Trauma Stress*. 2018;31(6):886-898. doi:10.1002/jts.22338.

116. McGuire Stanbury TM, Drummond PD, Laugharne J, Kullack C, Lee CW. Comparative Efficiency of EMDR and Prolonged Exposure in Treating Posttraumatic Stress Disorder: A Randomized Trial. *J EMDR Prac Res*. 2020;14(1):2-12. doi:10.1891/1933-3196.14.1.2.

117. Stenmark H, Catani C, Neuner F, Elbert T, Holen A. Treating PTSD in refugees and asylum seekers within the general health care system. A randomized controlled multicenter study. *Behav Res Ther*. 2013;51(10):641-647. doi:10.1016/j.brat.2013.07.002.

118. Surís A, Link-Malcolm J, Chard K, Ahn C, North C. A randomized clinical trial of cognitive processing therapy for veterans with PTSD related to military sexual trauma. *J Trauma Stress*. 2013;26(1):28-37. doi:10.1002/jts.21765.

119. Taylor S, Thordarson DS, Maxfield L, Fedoroff IC, Lovell K, Ogrodniczuk J. Comparative efficacy, speed, and adverse effects of three PTSD treatments: exposure therapy, EMDR, and relaxation training. *J Consult Clin Psychol*. 2003;71(2):330-338. doi:10.1037/0022-006x.71.2.330.

120. Ter Heide, F Jackie June, Mooren TM, van de Schoot R, Jongh A de, Kleber RJ. Eye movement desensitisation and reprocessing therapy v. stabilisation as usual for refugees: randomised controlled trial. *Br J Psychiatry*. 2016;209(4):311-318. doi:10.1192/bjp.bp.115.167775.

121. Thorp SR, Glassman LH, Wells SY, et al. A randomized controlled trial of prolonged exposure therapy versus relaxation training for older veterans with military-related PTSD. *J Anxiety Disord*. 2019;64:45-54. doi:10.1016/j.janxdis.2019.02.003.

122. Tylee DS, Gray R, Glatt SJ, Bourke F. Evaluation of the reconsolidation of traumatic memories protocol for the treatment of PTSD: a randomized, wait-list-controlled trial. *Journal of Military, Veteran and Family Health*. 2017;3(1):21-33. doi:10.3138/jmvfh.4120.

123. van den Berg, David P G, de Bont, Paul A J M, van der Vleugel, Berber M, et al. Prolonged exposure vs eye movement desensitization and reprocessing vs waiting list for posttraumatic stress disorder in patients with a psychotic disorder: a randomized clinical trial. *JAMA Psychiatry*. 2015;72(3):259-267. doi:10.1001/jamapsychiatry.2014.2637.

124. van der Kolk, Bessel A, Spinazzola J, Blaustein ME, et al. A randomized clinical trial of eye movement desensitization and reprocessing (EMDR), fluoxetine, and pill placebo in the treatment of posttraumatic stress disorder: treatment effects and long-term maintenance. *J Clin Psychiatry*. 2007;68(1):37-46. doi:10.4088/jcp.v68n0105.

125. van der Kolk, Bessel A, Stone L, West J, et al. Yoga as an adjunctive treatment for posttraumatic stress disorder: a randomized controlled trial. *J Clin Psychiatry*. 2014;75(6):65. doi:10.4088/JCP.13m08561.

126. van Gelderen MJ, Nijdam MJ, Haagen JFG, Vermetten E. Interactive Motion-Assisted Exposure Therapy for Veterans with Treatment-Resistant Posttraumatic Stress Disorder: A Randomized Controlled Trial. *Psychother Psychosom*. 2020;89(4):215-227. doi:10.1159/000505977.

127. Vaughan K, Armstrong MS, Gold R, O'Connor N, Jenneke W, Tarrier N. A trial of eye movement desensitization compared to image habituation training and applied muscle relaxation in post-traumatic stress disorder. *J Behav Ther Exp Psychiatry*. 1994;25(4):283-291. doi:10.1016/0005-7916(94)90036-1.

128. Wagner AW, Jakupcak M, Kowalski HM, Bittinger JN, Golshan S. Behavioral Activation as a Treatment for Posttraumatic Stress Disorder Among Returning Veterans: A Randomized Trial. *Psychiatr Serv*. 2019;70(10):867-873. doi:10.1176/appi.ps.201800572.

129. Wahbeh H, Goodrich E, Goy E, Oken BS. Mechanistic Pathways of Mindfulness Meditation in Combat Veterans With Posttraumatic Stress Disorder. *J Clin Psychol*. 2016;72(4):365-383. doi:10.1002/jclp.22255.

130. Wells A, Colbear JS. Treating posttraumatic stress disorder with metacognitive therapy: a preliminary controlled trial. *J Clin Psychol*. 2012;68(4):373-381. doi:10.1002/jclp.20871.

131. Wells A, Walton D, Lovell K, Proctor D. Metacognitive Therapy Versus Prolonged Exposure in Adults with Chronic Post-traumatic Stress Disorder: A Parallel Randomized Controlled Trial. *Cogn Ther Res*. 2015;39(1):70-80. doi:10.1007/s10608-014-9636-6.

132. Yurtsever A, Konuk E, Akyüz T, et al. An Eye Movement Desensitization and Reprocessing (EMDR) Group Intervention for Syrian Refugees With Post-traumatic Stress Symptoms: Results of a Randomized Controlled Trial. *Front Psychol*. 2018;9:493. doi:10.3389/fpsyg.2018.00493.

133. Zang Y, Hunt N, Cox T. A randomised controlled pilot study: the effectiveness of narrative exposure therapy with adult survivors of the Sichuan earthquake. *BMC Psychiatry*. 2013;13:41. doi:10.1186/1471-244X-13-41.

134. Zang Y, Hunt N, Cox T. Adapting narrative exposure therapy for Chinese earthquake survivors: a pilot randomised controlled feasibility study. *BMC Psychiatry*. 2014;14:262. doi:10.1186/s12888-014-0262-3.

135. Zlotnick C, Shea TM, Rosen K, et al. An affect-management group for women with posttraumatic stress disorder and histories of childhood sexual abuse. *J Trauma Stress*. 1997;10(3):425-436. doi:10.1023/a:1024841321156.

136. Zoellner LA, Telch M, Foa EB, et al. Enhancing Extinction Learning in Posttraumatic Stress Disorder With Brief Daily Imaginal Exposure and Methylene Blue: A Randomized Controlled Trial. *J Clin Psychiatry*. 2017;78(7):e782-e789. doi:10.4088/JCP.16m10936.

**Appendix D. Number of active and control conditions examined in the included studies**

| Comparisons | | Post | FU1 | FU2 |
| --- | --- | --- | --- | --- |
| All active conditions vs. | PCC | 77 | 21 | 8 |
|  | ACC | 59 | 30 | 19 |
| TF-CBT vs. | PCC | 47 | 13 | 6 |
|  | ACC | 33 | 20 | 17 |
|  | EMDR | 10 | 4 | n.a. |
|  | OAC | 19 | 11 | 11 |
| EMDR vs. | PCC | 7 | n.a. | n.a. |
|  | ACC | 11 | n.a. | n.a. |
| Other active conditions (OAC) vs. | PCC | 21 | 4 | n.a. |
|  | ACC | 15 | 7 | n.a. |

Abbreviations. ACC = Active Control Condition, included Treatment as Usual (TAU), supportive counseling, active listening, guided psychoeducation, medical placebo, stress inoculation training, self-help booklets and guided relaxation training; EMDR = Eye Movement Desensitization and Reprocessing; FU = Follow-up; n.a. = number of trials too small (*k* ≤ 3) to conduct analysis; OAC = Other Active Conditions; TF-CBT = Trauma-Focused Cognitive Behavior Therapy.

**Appendix E. Efficacy of psychological interventions including outlier- and asymmetry-adjustments**

| **Post** | | | | | | | **FU** | | | | |  |  |
| --- | --- | --- | --- | --- | --- | --- | --- | --- | --- | --- | --- | --- | --- |
|  | ***k*** | ***g*** | **SE** | **95% CI (PI)** | ***I^2^*** | **NNT** | ***FU1 vs FU2*** | ***k*** | ***g*** | **SE** | **95% CI (PI)** | ***I^2^*** | **NNT** |
| **Overall outcomes** | | | | | | | | | | | |  |  |
| All active conditions vs PCC | 77 | 1.09*** | 0.08 | 0.93; 1.25  (-0.13; 2.31) | 81.22*** | 1.79 | 1 | 21 | 0.81*** | 0.12 | 0.56; d1.05  (-0.15; 1.77) | 73.47*** | 2.32 |
| (outlier-adjusted^a)^ | 73 | 0.99*** | 0.07 | 0.86; 1.13  (0.02; 1.96) | 73.86*** | 1.93 | 2 | 8 | 0.67*** | 0.08 | 0.51; 0.83  (0.47; 0.88) | 8.14 | 2.73 |
| All active conditions vs ACC | 59 | 0.47*** | 0.06 | 0.35; 0.58  (-0.17; 1.11) | 57.23*** | 3.86 | 1 | 30 | 0.47*** | 0.09 | 0.31; 0.64  (-0.22; 1.17) | 57.26*** | 3.80 |
| (asymmetry-adjusted^b)^ | 63 | 0.42*** | 0.06 | 0.30; 0.54  (n.a.)^c^ | 63.53*** | 4.27 | 2  (asymmetry-adjusted) | 19 | 0.78*** | 0.12 | 0.54; 1.03  (-0.09; 1.65) | 68.09*** | 2.38 |
|  |  |  |  |  |  |  |  | 24 | 0.57*** | 0.14 | 0.31; 0.84  (n.a.) | 78.11*** | 3.17 |
| **Subgroup analyses** | | | | | | | | | | | |  |  |
| TF-CBT vs PCC  (outlier-corrected) | 47 | 1.23*** | 0.12 | 1.00; 1.47  (-0.24; 2.71) | 85.90*** | 1.62 | 1 | 13 | 0.89*** | 0.17 | 0.56; 1.22  (-0.18; 1.97) | 77.43*** | 2.12 |
|  | 45 | 1.14*** | 0.11 | 0.93; 1.35  (-0.11; 2.40) | 81.99*** | 1.72 | 2 | 6 | 0.75*** | 0.09 | 0.57; 0.93  (0.56; 0.95) | 2.80 | 2.47 |
| TF-CBT vs ACC | 33 | 0.49*** | 0.08 | 0.33; 0.66  (-0.24; 1.23) | 61.71*** | 3.68 | 1 | 20 | 0.57*** | 0.10 | 0.38; 0.76  (-0.02; 1.16) | 45.03* | 3.19 |
|  |  |  |  |  |  |  | 2 | 17 | 0.79*** | 0.14 | 0.52; 1.06  (-0.15; 1.73) | 70.21*** | 2.36 |
| TF-CBT vs OAC | 19 | 0.08 | 0.06 | -0.03; 0.20  (0.04; 0.22) | 33.47* | 20.94 | 1 | 11 | 0.22* | 0.09 | 0.05; 0.39  (-0.21; 0.64) | 53.60* | 8.22 |
| (outlier-adjusted) | 18 | 0.07 | 0.06 | -0.04; 0.18  (-0.20; 0.34) | 31.39 | 25.05 | 2  (outlier-adjusted) | 11 | 0.15** | 0.06 | 0.04; 0.26  (0.04; 0.26) | 0.00 | 11.65 |
|  |  |  |  |  |  |  |  | 9 | 0.10 | 0.06 | -0.01; 0.22  (-0.01; 0.22) | 0.00 | 16.91 |
| TF-CBT vs EMDR | 10 | -0.06 | 0.21 | -0.48; 0.35  (-1.21; 1.09) | 70.79** | -28.97 | 1 | 4 | -0.12 | 0.30 | -0.72; 0.47  (-1.18; 0.94) | 54.29 | -14.32 |
|  |  |  |  |  |  |  | 2 | n.a. (*k* = 2) | | | | | |
| EMDR vs PCC | 7 | 1.19*** | 0.22 | 0.76; 1.62  (0.16; 2.22) | 71.86*** | 1.67 | 1 & 2 | n.a. (*k* = 2); n.a. (*k* = 1), respectively | | | | | |
| EMDR vs ACC | 11 | 0.42*** | 0.10 | 0.22; 0.62  (0.15; 0.68) | 7.31 | 4.29 | 1 & 2 | n.a. (*k* = 3); n.a. (*k* = 0), respectively | | | | | |
| Other active conditions (OAC) vs. PCC  (outlier-adjusted) | 21 | 0.76*** | 0.08 | 0.61; 0.91  (0.36; 1.16) | 30.33** | 2.45 | 1 | 4 | 0.41** | 0.13 | 0.14; 0.67  (0.14; 0.67) | 0.00 | 4.39 |
|  | 20 | 0.73*** | 0.07 | 0.60; 0.87 (0.46; 1.00) | 15.17 | 2.53 | 2 | n.a. (*k* = 1) | | | | | |
| Other active conditions (OAC) vs. ACC  (asymmetry-adjusted) | 15 | 0.53*** | 0.13 | 0.27; 0.79  (-0.36; 1.42) | 73.77*** | 3.42 | 1 | 7 | 0.49 | 0.21 | 0.09; 0.90  (-0.50; 1.49) | 73.56** | 3.67 |
|  | 17 | 0.44** | 0.14 | 0.17; 0.72 (n.a.) | 76.77*** | 4.09 | 2 | n.a. (*k* = 2) | | | | | |

Abbreviations. ACC = Active Control Conditions; *k* = number of trials included in the analysis for the given comparison; n.a. = number of trials too small (*k* < 4) to conduct analysis; EMDR = Eye Movement Desensitization and Reprocessing; FU = Follow-up; OAC = Other Active Conditions; PCC = Passive Control Conditions; PI = prediction interval; TF-CBT = Trauma-focused Cognitive Behavior Therapy; WL = waitlist.

* *p* < .05, ** *p* < .01, *** *p* < .001.

^a^Only if applicable (i.e., *k* ≥ 10 and at least one outlier present).
^b^Only if applicable (i.e., *k* ≥ 10 and at least one additional study introduced by the trim and fill method).

^c^The trim and fill method does not supply prediction intervals.

**Appendix F. Comparison of high quality (HQ) and lower quality trials (Other) when compared to passive control conditions at posttreatment including outlier- and asymmetry-adjustments**

|  |  |  | ***k*** | ***g*** | **95% CI**  **(PI)** | | ***I*²** | **NNT** | ***p*** | |
| --- | --- | --- | --- | --- | --- | --- | --- | --- | --- | --- |
| High quality (HQ) | | HQ | 12 | 0.87*** | 0.61; 1.14  (0.03; 1.72) | | 77.05*** | 2.16 | .233 | |
|  |  | Other  (outlier-adjusted) | 65 | 1.14*** | 0.96; 1.33  (-0.19; 2.47) | | 81.34*** | 1.72 |  |  |
|  |  |  | 63 | 1.08*** | 0.91; 1.24  (-0.08; 2.23) | | 77.03*** | 1.81 |  | |
| CAPS only | | HQ | 8 | 0.78*** | 0.48; 1.07  (0.03; 1.52) | | 70.75** | 2.40 | .098 | |
|  |  | Other  (outlier-adjusted | 32 | 1.21*** | 0.96; 1.45  (-0.05; 2.47) | | 80.44*** | 1.65 |  |  |
|  |  |  | 31 | 1.14*** | 0.92; 1.36  (0.07; 2.20) | | 75.08*** | 1.73 |  | |
| Type of treatment | TF-CBT | HQ | 8 | 0.83*** | 0.54; 1.13  (0.08; 1.59) | | 71.47** | 2.25 | .119 | |
|  |  | Other | 39 | 1.33*** | 1.05; 1.61  (-0.28; 2.94) | | 85.77*** | 1.53 |  |  |
|  | EMDR | HQ | n.a. (*k* = 2) | | | | | | n.a. | |
|  |  | Other | 5 | 1.16*** | 0.70; 1.61  (0.29; 2.02) | | 52.97 | 1.70 |  |  |
|  | Other | HQ | n.a. (*k* = 2) | | | | | | n.a. | |
|  |  | Other  (outlier-adjusted)  (asymmetry-adjusted) | 19 | 0.78 | 0.60; 0.97  (0.24; 1.32) | | 42.93** | 2.38 |  |  |
|  |  |  | 18 | 0.74 | 0.58; 0.90  (0.36; 1.13) | | 62.29*** | 2.50 |  | |
|  |  |  | 21 | 0.72 | 0.49; 0.94  (n.a.) | | 62.42*** | 2.58 |  | |
| Treatment format | Individual | HQ | 12 | 0.87*** | 0.61; 1.14  (0.03; 1.72) | | 77.05*** | 2.16 | .128 | |
|  |  | Other | 51 | 1.24*** | 1.02; 1.47  (-0.21; 2.70) | | 83.27*** | 1.61 |  |  |
|  | Group | HQ | n.a. (*k* = 0) | | | | | | - | |
|  |  | Other  (asymmetry-adjusted) | 14 | 0.84*** | 0.56; 1.11  (-0.03; 1.70) | | 65.97*** | 2.24 |  |  |
|  |  |  | 18 | 1.03 | 0.75; 1.31  (n.a.) | | 73.91*** | 1.87 |  | |
|  | |  | ***k*** | **Intercept** | | ***b*** | ***I²*** | ***p*** | |  |
| Number of sessions as a continuous variable | | HQ | 12 | 0.79 | | 0.01 | 79.40*** | .928 | |  |
|  |  | Other  (outlier-adjusted) | 65 | 1.36 | | -0.02 | 81.41*** | .209 | |  |
|  |  |  | 63 | 1.20 | | -0.01 | 77.30 | .425 | |  |

Abbreviations. CAPS = Clinician-Administered PTSD Scale; *k* = number of trials included in the analysis for the given comparison; n.a. = not applicable (i.e., number of trials too small (*k* < 4) to conduct analysis); PCC = Passive Control Conditions; PI = Prediction Interval; TF-CBT = Trauma-focused Cognitive Behavior Therapy. *p*-values refer to the comparison of high quality vs. other studies and to the significance level of *b*.

* *p* < 0.05; ** *p* < 0.01 *** *p* < 0.001
